# Storing and analyzing a genome on a blockchain

**DOI:** 10.1186/s13059-022-02699-7

**Published:** 2022-06-29

**Authors:** Gamze Gürsoy, Charlotte M. Brannon, Eric Ni, Sarah Wagner, Amol Khanna, Mark Gerstein

**Affiliations:** 1 Program in Computational Biology and Bioinformatics, Yale University, Whitney Avenue, New Haven, CT 06520 USA; 2 Department of Molecular Biophysics and Biochemistry, Yale University, Whitney Avenue, New Haven, CT 06520 USA; 3 Current Address: Department of Biomedical Informatics, Columbia University, New York, NY USA; 4 Current Address: New York Genome Center, New York, NY USA; 5 Current Address: Stanford University, Stanford, CA USA; 6 Department of Computer Science, Yale University, Prospect Street, New Haven, CT 06520 USA; 7 Department of Biomedical Engineering, Johns Hopkins University, Baltimore, MD USA; 8 Department of Applied Mathematics, Johns Hopkins University, Baltimore, MD USA

**Keywords:** Blockchain, Multichain, Personal genome, Blockchain database

## Abstract

There are major efforts underway to make genome sequencing a routine part of clinical practice. A critical barrier to these is achieving practical solutions for data ownership and integrity. Blockchain provides solutions to these challenges in other realms, such as finance. However, its use in genomics is stymied due to the difficulty in storing large-scale data on-chain, slow transaction speeds, and limitations on querying. To overcome these roadblocks, we developed a private blockchain network to store genomic variants and reference-aligned reads on-chain. It uses nested database indexing with an accompanying tool suite to rapidly access and analyze the data.

**Supplementary Information:**

The online version contains supplementary material available at 10.1186/s13059-022-02699-7.

## Background

Modern advances in personalized medicine have resulted in an increasing number of individuals willing to sequence their own genome for disease-risk predictions and ancestry analysis, which has brought us closer to an era of genomic data-driven healthcare and biomedical research. Given the widespread interest in understanding one’s own genomic data, and the promise of genomic data for advancing biomedical research, it is almost inevitable that genome sequencing will become part of routine clinical care in the future and that the number of sequenced human genomes will continue to grow [1].

The increase in genetic testing in medical settings and through direct-to-consumer companies has raised questions related to data ownership. Data ownership is the possession and the responsibility of the information in the data, which implies both power and control. The control of the information is defined as the ability to access, create, modify, package, derive benefit from, sell, or remove the data and also the right to assign these access privileges to others [2]. For example, when an individual purchases a sequencing service from a company such as 23andMe, they are often giving that company the right to monetize their genomic data by selling it to pharmaceutical companies [3]. Ideally, individuals would retain ownership and be able to benefit from their own data. This enables them to control the right to share (privacy) and how to share (security) their own genetic data [4]. Yet, the storage and analysis infrastructure to achieve this goal is lacking. As a result, there is no technical solution to achieve the provenance and security of this highly sensitive, private, personal data such that data owners are empowered to contribute to science and medicine while keeping their data safe. Moreover, data ownership issues are not restricted to individual-level genomic data. There is an increase in human-derived functional genomic datasets, requiring attention in terms of who owns and controls these datasets.

There is increasing interest in blockchain technologies and their capabilities. Ongoing discussions have centered on how blockchain technology could change the currency of the world by addressing many challenges in the current financial systems at once (e.g., transaction speed, security, and removal of middle man). For example, non-fungible tokens (NFTs) are increasingly being used as a way to deploy valuable assets on public or hybrid blockchains. Moreover, blockchain technology is being used in many industries from supply chain management to the media and entertainment business. Naturally, there is interest in the capabilities of this technology in genomics as blockchain technology can establish verified and public proof of data ownership [5, 6]. However, no one has yet figured out how to store a large amount of data, such as the read stack observed in genome sequencing, in a blockchain. This is due to technical roadblocks posed by blockchain for large-scale data storage and analysis. In this study, for the first time, we implement an approach to store and analyze a genome in a blockchain.

Note, many up and coming biotech companies offer blockchain-based solutions for genomic data sharing (see Additional file 1 for more about these companies and blockchain technology). Because of the technical challenges related to storing genomes in a blockchain infrastructure, many blockchain genomics companies store the genomic data elsewhere, such as in Blockstack or the InterPlanetary File System (IPFS) [7, 8] and use blockchains as a log-keeping infrastructure (see Additional file 1 for more about the difference). In particular, IPFS is a decentralized file system that uses a distributed hash table for easy access to the shared files in an effort to create a resilient system for file storage and sharing.

The storage space and computational power required by blockchain is greater than a centralized database application due to the redundancy of storage and network verification protocols. The decentralized system also creates a higher latency (delay in data communication) during storage and retrieval of data. Additionally, transactions in the blockchain network require a cryptographic consensus verification, which makes them slow to publish data to the chain [9]. We propose that a carefully crafted private blockchain network can alleviate the problems related to both data ownership and control and technical difficulties. Moreover, it can maintain security and integrity of the data. As genomic data becomes increasingly integral to our understanding of human health and disease, its integrity and security must be a priority when providing solutions to storage and analysis. Corruption, change, or loss of personal genomes could create problems in patient care and research integrity in the future. An ideal implementation of personal genomic data storage would (1) protect from loss and manipulation, (2) provide appropriate access to clinicians and biomedical researchers, and (3) allow individuals control over their own genomic data. Private blockchains lack mathematical guarantees at the validation level, but still utilize the cryptographic safeguards and blockchain-specific data structures (e.g., Merkle trees) to make sure non-valid transactions are not added to the chain. Although proof-of-work is not used, transactions are verified using other consensus mechanisms. Private blockchain networks may be compared to distributed databases to illustrate their utility. Blockchain architecture has several advantages, as outlined by Kuo et al. [10]. Briefly, blockchain excels in decentralized database management, immutability (provided by the hash of a new block containing the information of the blocks that come before it), data provenance, robustness, security, and privacy. Additionally, a private blockchain may be converted into a consortium blockchain, in which consensus is controlled by a pre-selected set of nodes (i.e., proof of work in a limited way), further boosting its security.

In this study, we present the first open-source, proof-of-concept private blockchain network, which allows efficient storage and retrieval of personal genetic variants [often stored as variant call format (VCF) files] and raw genomic reads [often stored as sequence alignment map (SAM) files] [11, 12]. To overcome the challenges described above, we developed novel data structures based on nested database indexing, file-format modifications, and compression techniques with the open-source blockchain API MultiChain. We made use of their “data stream” feature, which allows users to create multiple key-value, time-series, or identity databases that can be used for data sharing, time-stamping, and encrypted archiving (see Additional file 1). We provide two modules. The first module is a lightweight software that allows users to efficiently store (VCFChain) and query (VCFtool) VCF files. The second module is more resource intensive and allows users to create a chain and insert SAM data into it (SAMchain) and perform a selection of functions (SCtools) such as querying, depth analysis, pile-ups for variant calling, and re-creating SAM files and their derivatives (such as BAM and CRAM files).

Because the process of pushing the data into a blockchain is done once but querying and analyzing the data is a continuous process, we focused on optimizing the data storage towards faster querying/analysis with multiple levels of indexing schemes. This is different than the current blockchain genomic data storage options introduced in private industry, where the data storage is optimized, hence providing no option for querying and analyzing the data.

## Results

### Private blockchain networks can help with control of data

**Fig. 1:**
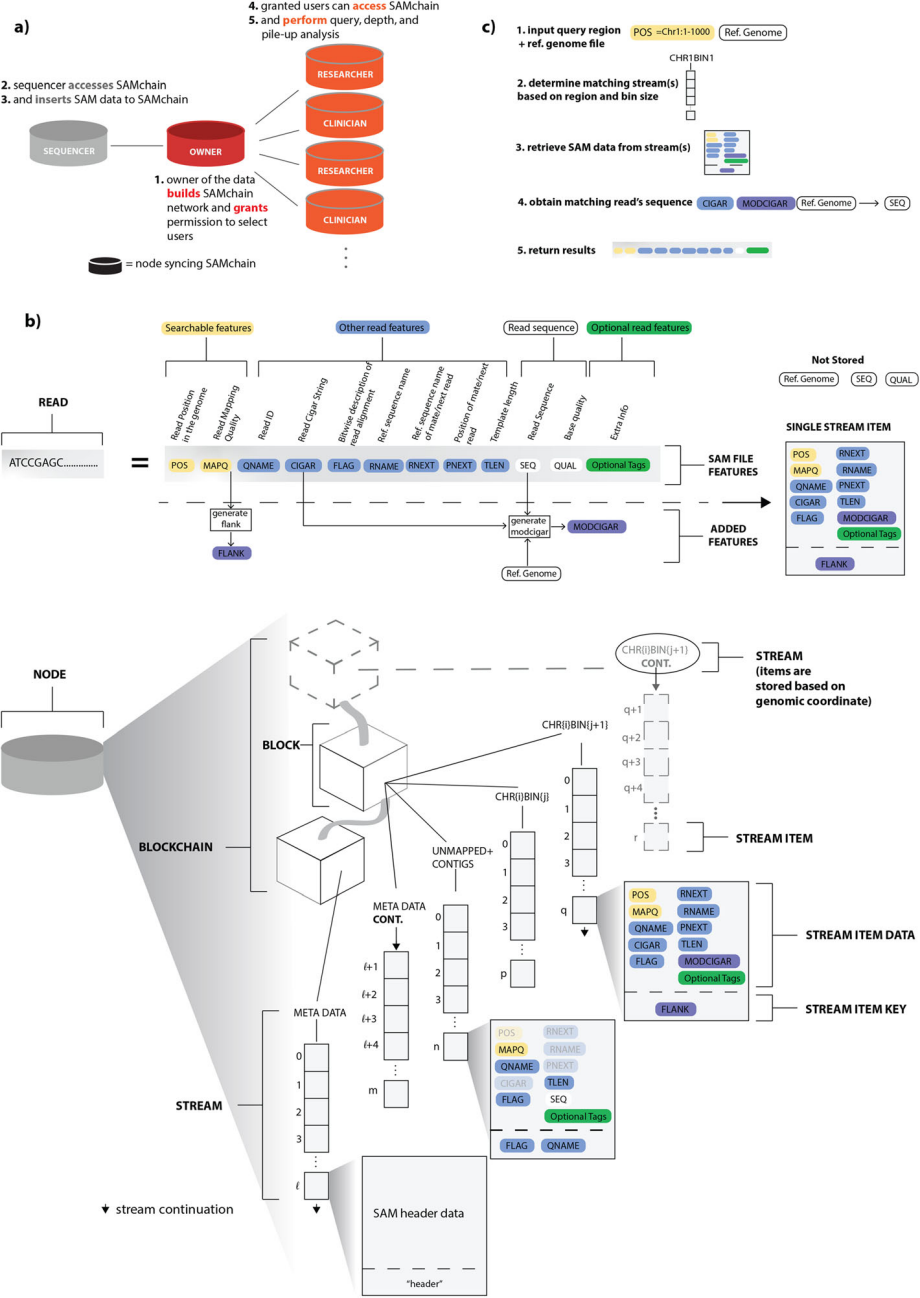
SAMchain design and implementation. **a** Overview of the SAMchain network ecosystem. The network consists of owner, sequencer, clinician, and researcher nodes. The owner node builds the SAMchain and the sequencer node accesses the chain and inserts SAM data into it. The clinician and researcher nodes access the SAMchain and analyze the on-chain SAM data. **b** Details of data storage in SAMchain. A read is typically stored in a SAM file containing several features. Our data structure is organized by genomic location. A single stream, named metaData, contains all of the header data and other chain info. Many other streams serve as bins by genomic location and hold the SAM feature data and MODCIGAR. A FLANK feature is used to indicate whether a read’s position spans two consecutive bins. Stream items correspond to a single read. A single stream, named unmappedANDcontigs stores unmapped reads and contigs. **c** Overview of the query process. Upon querying a genomic location, our algorithm searches through the binned streams to obtain the SAM data and MODCIGAR features corresponding to the specified location. These data, in combination with a reference genome, yield a complete SAM read. Our algorithms and stream-based data structures are built on top of MultiChain, which provides the underlying blockchain, stream design, and network configuration

We envisioned a network of sequencers, owners, clinicians, and researchers, each syncing VCFchain or SAMchain (Fig. 1a). The owner node initializes a VCFchain or SAMchain, including the data streams that store the VCF or SAM data. The sequencer node generates the VCF/SAM data and requests access to the data owner’s VCFchain or SAMchain. The owner grants access, allowing the sequencer to push the owner’s data to their chain. The clinician and researcher nodes, upon making contact with the owner through other means, may also request access to the owner’s VCFchain or SAMchain and make use of the VCFtools or SCtools modules to analyze the data. In this scheme, the owner may change the permissions of VCFchain or SAMchain at any time. MultiChain, the technology that SAMChain is built upon, provides access control, in which the owner of the blockchain network can grant permission to a user and make the user part of the blockchain network. It also provides the option of granting only partial access to the chain. Moreover, every time a user subscribes to the network and pushes data to the network, it is recorded in the blockchain with precise timestamps.

### SAMchain’s design provides advantages over traditional blockchain data storage methods

Next, we considered how best to configure data storage in SAMchain. The naive way to store data in a bitcoin-like blockchain would be to append small amounts of data to each transaction using OP_RETURN, a script opcode allowing the sender to send a small amount of data (which varies between platforms) with their transaction. The OP_RETURN data are mined into a block along with the rest of the transaction. The data that are pushed using OP_RETURN are indexed in a transaction with a unique identifier, often called “ref” in a transaction. Each transaction can hold data around ~ 80 bytes, which means genomic data must be stored within numerous transactions. The unique identifiers must be stored somewhere separately, as one can retrieve data only with ref. As one can imagine, querying the genome with a specific position, or doing a pile-up analysis with OP_RETURN, is extremely difficult and inefficient due to the lack of required data structures [13]. In addition to storing the data embedded in the transactions, MultiChain offers another data structure to store the data on the chain, called “data streams.” Each stream item is represented by a blockchain transaction, which is mined and validated by every node like any other transaction (please see MultiChain white paper for details at https://www.multichain.com/download/MultiChain-White-Paper.pdf  [last access June, 2022]). The embedded key:value property of the streams allows for efficient retrieval of the data on the chain, because when a node in a MultiChain network subscribes to a stream, it indexes the stream’s content in real time in order to enable efficient retrieval by the keys (see https://www.multichain.com/developers/data-streams/ for details [last access June, 2022]). Note that blocks are created and sealed based on the transaction (e.g., data storage) timestamps. Depending on the timestamp that the data was pushed to the chain, a single data stream can be stored over multiple blocks or a single block might store multiple data streams.

The naive way to store data in a MultiChain stream would be to push all the data to a single stream, and then query from it. However, the query algorithm must check each item to determine if it matches the queried position or range of positions. Were each genomic read defined by a single point position, we would not have this problem; we would simply store point positions as keys. However, because a read is defined by a range of positions, with overlap between reads, our query algorithm must check that a given read overlaps with the range or point queried. This is not possible in a traditional key:value-stored dictionary (i.e., streams in MultiChain). To achieve this kind of query with efficiency, we created streams binned by genomic location (Fig. 1b,c). This increases the time it takes to create a SAMchain (which is only done once), as many streams must be created, but significantly decreases the query time, making SAMchain a viable way of storing raw genomic reads on a blockchain. In Table 1, we summarize the contributions of MultiChain to our methods vs. those of SAMchain itself.

**Table 1:**
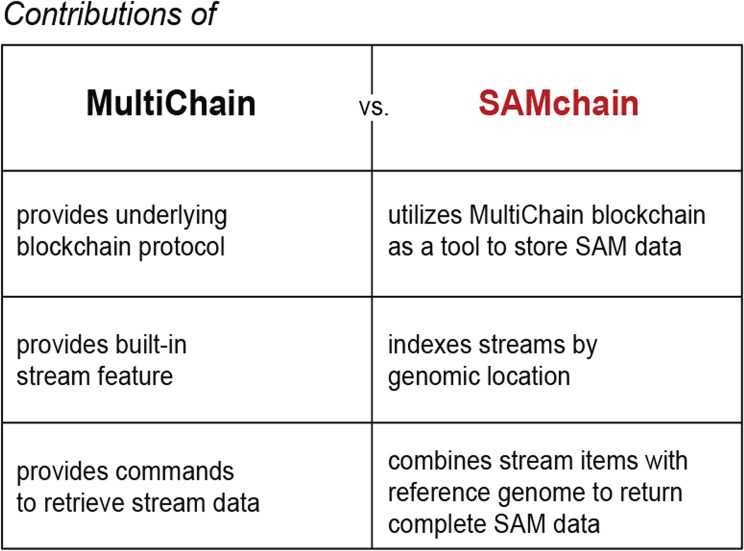
Contributions of MultiChain vs. SAMchain. The novel method presented in this paper is SAMchain, a tool that is built upon the functionality of the MultiChain blockchain platform/API. Here we summarize how the contributions of SAMchain differ from those of MultiChain

### SAMchain provides a platform for storing next-generation sequencing (NGS) data in blockchain

**Fig. 2:**
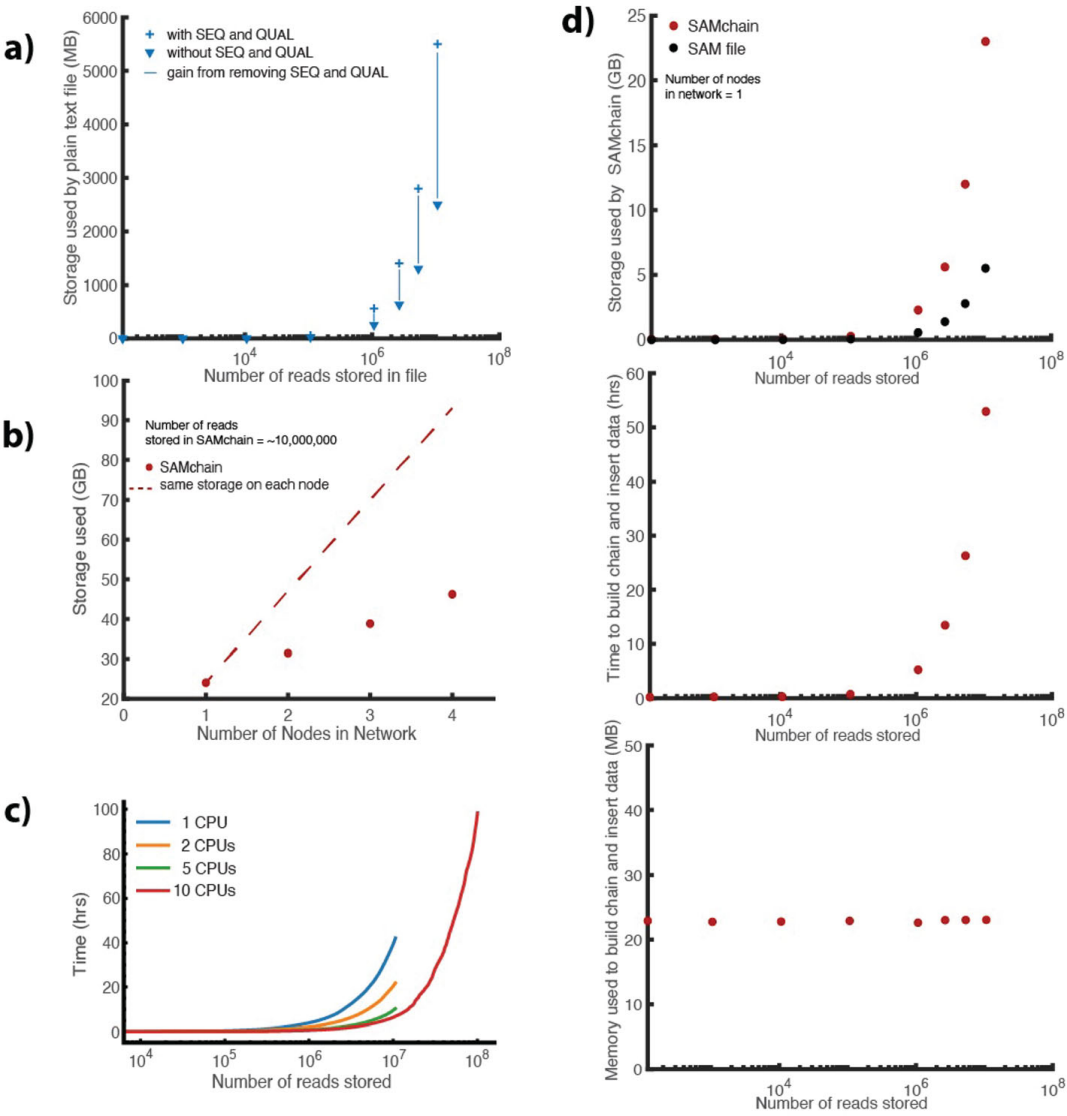
SAMchain performance. **a** Before inserting SAM data to a SAMchain, we remove the sequence and quality strings. In plain text, we measured the storage gain by removing these fields. **b** The total network storage required for a SAMchain storing ~ 10 million reads. **c** Total time to insert data to SAMchain as a function of number of reads when a different number of CPUs are used. **d** Storage (compared to a SAM file), time (for a single CPU), and memory used to build a SAMchain

**Fig. 3:**
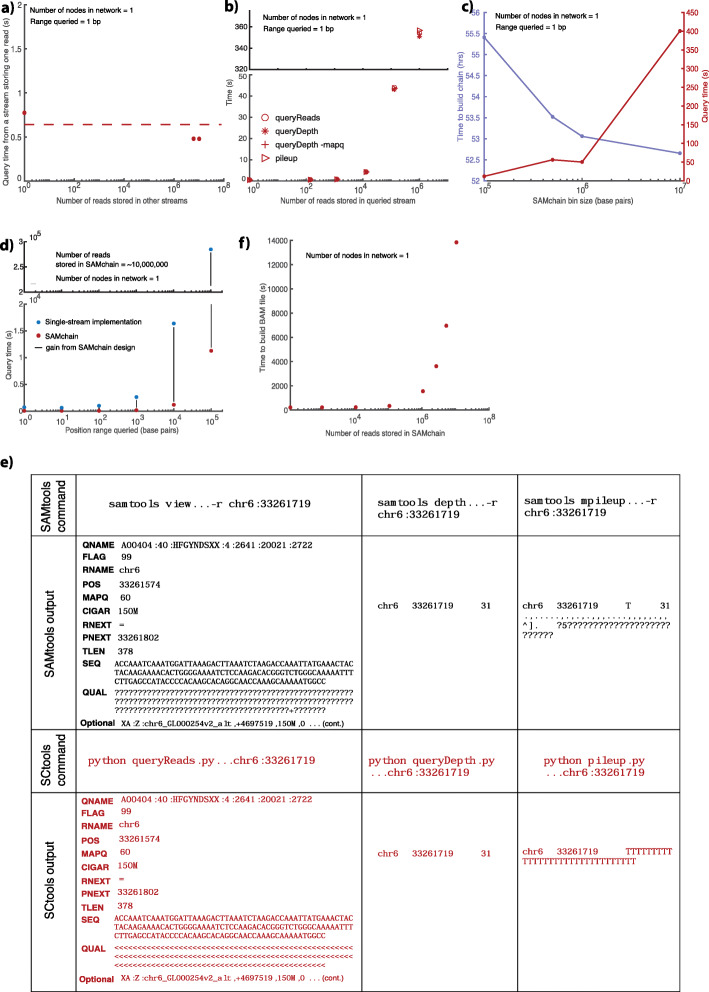
SCtools performance. **a** Query time from a MultiChain stream depends only on the number of entries in that stream, i.e., it is not affected by the number of reads stored in other streams in the chain. **b** In a single-node SAMchain network, we measured the time performance of queryReads, queryDepth, queryDepth and MAPQ, and pileup for 1-bp queries. Each module performs comparably, increasing linearly as the number of reads stored in the chain increases. **c** We measured the performance of queryReads for a SAMchain storing ~ 10,000,000 reads at different bin sizes. SAMchains with smaller bin sizes yield faster query times, but take longer to build. **d** We measured the effect of the range queried on performance time. Larger ranges are shown to yield longer query times. We showed the times of SAMchain (red) compared to a naive, single-stream implementation (blue). **e** We checked the output of each SCtools module compared to the equivalent SAMtools module

It is notoriously difficult to store large amounts of data in a blockchain, due to network latency and storage redundancy [10]. Thus, to store raw genomic data on-chain, we first needed to manipulate the information content for efficient storage. Whereas a SAM file stores a read’s sequence and the quality string of the read, SAMchain stores only the difference between a read and a reference genome. This design was inspired by the CRAM file format, in which a genomic reference file is optionally used to describe the difference between the aligned sequence and the reference sequence [14]. However, CRAM is a columnar file format composed of containers whereas SAMchain stores data in plain text. Our manipulation consists of storing a new data field that we refer to as the “modcigar,” a string containing the sequence data that differ from the reference (e.g., insertions). We show that there is a ~ 2-fold reduction in the storage with this manipulation (Fig. 2a). Figure 2b shows how adding nodes to the SAMchain private network affects the amount of data needed to be stored. Next, we designed the SAMchain data structure using MultiChain streams. As defined by MultiChain, streams are ordered lists of items, each with a publisher (who digitally signed the item), a set of keys (to be used for retrieval), some data (which are embedded on-chain in a transaction), and some meta data (about the transaction and block corresponding to the item). As we discuss in the section above and show in Fig. 3b, querying data by genomic position from a single stream would be very time-inefficient. To address this problem, our code creates several streams binned by genomic position based on an input bin size. For example, the length of human chromosome 1 (build GRCh38) is 248,956,422 base pairs. If the user were to set the bin size to one million base pairs, then 248,956,422/1,000,000 = 249 streams would be created for chromosome 1, named chr1stream{j}, where *j* ranges from 1 to 249. In these streams, we stored the SAM features in the data field, and a feature called “flank” in the key field, which indicates whether a read’s coordinates span one stream (flank = 0) or two (flank = 1) (see Additional file 1: Fig. S1). The flank feature is necessary because the position of some reads will naturally span two consecutive streams. This design allows our query algorithm to know exactly which streams to search during a query, and to search through streams with fewer items. We confirmed that the retrieval time in MultiChain is based only on the number of entries in a given stream (and unaffected by other streams on-chain) (Fig. 3a). Our code also creates a metaData stream to store the file header, and an unmappedANDcontigs stream to store any unmapped reads and contigs in the case that they can be realigned in the future (see Additional file 1: Table S1). The design of the SAMchain data structure is shown in Fig. 1b.

We measured the time, memory, and storage requirements of building a SAMchain. All the tests described in the “Results” were completed using an alignment file curated from the high-coverage whole-genome sequencing data of individual NA12878 from the 1000 Genomes Project as input data. For speedy testing, we constructed a SAM file with one disease-causing locus of roughly 1–5 million base pairs per chromosome. The details of these loci are provided in Additional file 1: Table S2.

For a fixed bin size (1 million base pairs) and varying number of input reads, we evaluated the time, storage, and memory requirements of building and inserting data into a SAMchain. As shown in Fig. 2c, the storage required per node by SAMchain is approximately 5-fold greater than that required by a SAM file. For example, our input SAM file containing ~ 10 million reads requires 5.5 GB. Building the corresponding SAMchain in a one-node network requires ~ 25 GB. As shown in Fig. 2b, the storage requirements of a SAMchain increase with increasing nodes in the network, as each node stores redundant data. For a chain storing ~ 10 million reads, each additional node requires 7.4 GB. The node that inserts the SAM data into the chain requires the most storage because MultiChain keeps a “wallet” directory, which stores transaction data especially relevant to the local node (see Additional file 1 details). In Fig. 2d, we measured the time and memory it takes to build and insert data into a SAMchain as a function of the number of reads in the input SAM file, which show linear and constant trends, respectively. To build and insert 10,000,000 reads to a SAMchain, it takes ~ 55 h in a single node. However, keep in mind that pushing data to SAMchain must only be done once.

Since each read in a SAM file is independent and the streams to store the reads are automatically determined by our algorithm based on its coordinates, we can use multiple nodes to push the data in a SAM file in a parallel fashion. To this end, we parallelized our codebase and showed that we can improve data insertion time by 5-fold using 10 CPUs at the same time (Fig. 2c). We also found that the 5-fold increase is the limit of the possible speedup because of the transaction latency in the blockchain. (This latency is due to safeguards such as proof of authority.) As an illustration, we tried to push 100,000,000 reads into the SAMchain, which took ~ 100 h using 10 CPUs. Note that there is a balance between how much data is stored and how quickly the data can be queried. As the data storage is needed only once, our optimizations are geared towards efficient queries. This is different than current blockchain genomic data storage options, where the data storage is optimized and outsourced to other distributed systems such as IPFS; hence, there is no option for querying and analyzing the data on the chain.

### SCtools can query NGS properties directly from SAMchain

We developed SCtools to query reads and other NGS properties from a SAMchain. Specifically, we developed four modules: queryReads, queryDepth, pileup, and buildBAM. queryReads queries a SAMchain based on one of four SAM features and outputs reads in SAM format. queryDepth performs depth analysis and pileup performs pileup analysis on an input range of genomic coordinates. buildBAM reconstructs a BAM file from a SAMchain. To evaluate the scalability and performance of these modules, we first showed that the time to perform a point query depends only on the number of reads in the queried stream and is not affected by reads stored in other on-chain streams (Fig. 3a). Next, we measured the time requirements of each SCtools module as a function of the number of reads stored in the queried stream (Fig. 3b). The modules show comparable time efficiency, increasing linearly with the number of reads stored in the queried stream (Fig. 3b). We also checked the effect of “AND” queries, that is, filtering a depth query by MAPQ score (Fig. 3b, “+” icon). We found that this type of query performed just as well as the other modules. In Fig. 3c, we investigated the impact of changing the SAMchain bin size for a fixed number of reads stored (~ 10 million). We found that increasing the bin size increases the query time, which we expected because a larger bin size also contains a higher number of reads relative to a smaller bin size. However, increasing the bin size also decreases the total number of streams, which reduces the time required to build the SAMchain (Fig. 3c). In Fig. 3d, we measured query time for a SAMchain storing a fixed number of reads (~ 10 million) as a function of the range of genomic coordinates queried. We found that query time increases linearly with an increasing range queried. This shows that if we increase the bin sizes, we can reduce storage size by ~ 6% of the original storage need (55.5 GB vs. 52.5 GB). On the other hand, the time to query data becomes ~ 40-fold slower (10 s vs. 400 s). These trade-offs can help dictate whether a particular use case is appropriate for SAMchain. In Fig. 3e, we show the output of each SCtools module compared to that of the comparable SAMtools function. In Fig. 3f, we measured the time it takes to build a BAM file from the read data stored in a SAMchain. The time increases linearly with an increasing number of reads stored in the chain.

As can be seen in Fig. 3b, the time it takes to query a single genomic location using our different query mechanisms in a network with 10 million reads divided into 10,000 reads/stream is around 0.5 s. Note that as was shown in Fig. 3a, the query time depends on the stream size; that is, if we have 10,000 reads per stream with 500 million reads, the query still takes 0.5 s because of the nested indexing scheme. As a comparison, we ran various queries with plaintext tool SAMtools [11] using a BAM file with ~ 60 million reads. We found that pile up for a single location takes about 0.05 s; querying a depth of a single location takes about 0.02 s; and querying the reads mapped to a single location takes about 0.08 s. This shows that SCtools, on average, is about 10 times slower than SAMtools due to the added security. However, note that, in order to be able to perform these queries with SAMtools, one needs to sort and index the BAM files first, which is known to be a slow process. In SAMchain, reads are already stored in an indexed way based on their genomic coordinates, and therefore, there is no need for such pre-processing. However, one can think of sorting process as analogous to pushing the SAM data to the blockchain network as both processes are slow, but done only once.

While we evaluated the performance using DNA sequencing data, SAMchain and SCtools are compatible with any NGS data, including functional genomic data. For example, SCtools can be utilized to query variants in a gene or the depth distribution of exons of interest on raw RNA sequencing reads.

### VCFChain and VCFquery modules provide faster options for direct query of genomic variants

**Fig. 4:**
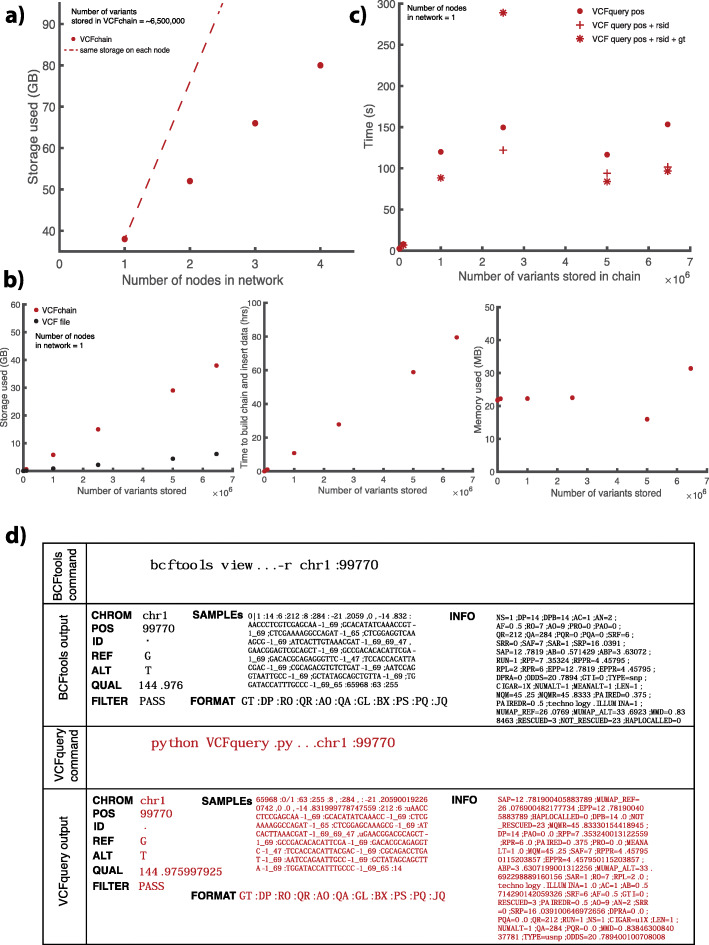
VCFchain and query performance. **a** The total network storage used by a VCFchain (storing 6,458,146 variants) as a function of the number of nodes in the network. **b** The storage, time, and memory requirements of building and inserting data to a VCFchain in a single-node network as a function of the number of reads stored in the chain. Storage is compared to that used by a VCF file. **c** The time requirements for VCFquery as a function of the number of reads stored in the chain, compared to the retrieval time from a VCF file using BCFtools. **d** The output of VCFquery compared to that of BCFtools for a given read

There is significant value in storing and sharing aligned genomic reads, as opposed to just the genomic variants called from the aligned reads. Access to raw reads is important because if a new reference genome build is available, the reads can be realigned and new variants may be observed, thereby maximizing the utility of the data. Thus, our primary goal was to create a blockchain storage system for SAM files. However, practical applications of SAMchain and SCtools can be limited due to the large data sizes originating from the redundancy in blockchain. There can also be value in sharing variant data (VCF files). Thus, we also developed VCFchain and a VCF query module. VCFchain stores data from a VCF file on-chain, and VCF query can query variants from the chain by genomic position, along with reference single-nucleotide polymorphism (SNP) ID (rsID) and/or genotype. To test these modules, we used a VCF file from a consented individual in the ENCODE data portal. We chose this VCF because it contains not only SNPs and small insertions and deletions but also a full set of structural variants. In Fig. 4, we show the performance of building VCFchain and VCFquery. As shown in Fig. 4a, the storage required for a one-node network per node by a VCFchain storing ~ 6.5 million variants is approximately 40 GB. As more nodes are added to the network, the total storage increases. In this case, each additional node requires 14 GB. In Fig. 4b, we measured the storage, time, and memory requirements of VCFquery in a one-node network with an increasing number of stored variants. In Fig. 4c, we measured the time requirements of VCFquery for position queries, along with position + rsID and position + rsID + genotype queries. Figure 4d shows the output of VCFquery for a given read compared to that of BCFtools.

Our VCFchain infrastructure could also be used to store and share variants from a cohort of individuals. For example, one could build upon our code base to create a VCFchain with the somatic variants of The Cancer Genome Atlas dataset stratified by cancer type.

More technical details on SAMchain, Sctools, VCFchain, and VCFtools can be found in Additional file 1.

### SAMchain is the first proof-of-concept framework to store raw genomic reads on a blockchain

In addition to comparing the performance of SAMchain and SCtools to that of SAMtools, we investigated the details of other genomic data storage platforms that use blockchain. Because the role of blockchain in these platforms is qualitatively different from that in SAMchain, we are not able to make quantitative comparisons. For example, storing links in a blockchain while storing the data elsewhere is fundamentally different than storing the data itself on-chain. Instead, we highlight the major differences between each platform and SAMchain in Table 2. Note that the information about each platform is derived from the limited knowledge provided by the company websites and whitepapers and is presented here to the best of our knowledge and their implementation as of February 2022. We identified four companies and/or projects that use blockchain in the context of genomic data storage: CrypDist, Zenome, Nebula Genomics, and the Cancer Gene Trust. Another is Encrypgen/Gene-Chain, but their limited documentation prevents any comparison with SAMchain. While each of these four platforms uses blockchain for some aspect of their network ecosystem, none uses it to store raw genomic reads on-chain. CrypDist stores links to data, which are stored in cloud buckets [15]. Zenome uses Ethereum Smart Contracts to facilitate transactions of genomic data, but stores the data off-chain in a distributed file storage system [7, 16]. Nebula Genomics uses Ethereum Smart Contracts to facilitate communication between nodes, and Blockstack to facilitate data storage, but Blockstack stores the data off-chain, either on a local drive or in the cloud. Users are then able to access these files using the bioinformatics analysis platform Arvados to analyze the data (Digital Ocean, S3, Dropbox) [8, 17]. Finally, the Cancer Gene Trust stores data off-chain in the IPFS and raw genomics data locally and uses Ethereum Smart Contracts to store references to the data files [7, 8, 18]. To the best of our knowledge, SAMchain is the first proof-of-concept framework to store raw genomic reads on a blockchain, on-chain. By embedding the data in the chain, SAMchain aims to preserve the integrity of the data by taking advantage of blockchain protocols designed to preserve the integrity of cryptocurrency transactions. Details of these platforms can be found in Additional file 1.

**Table 2:**
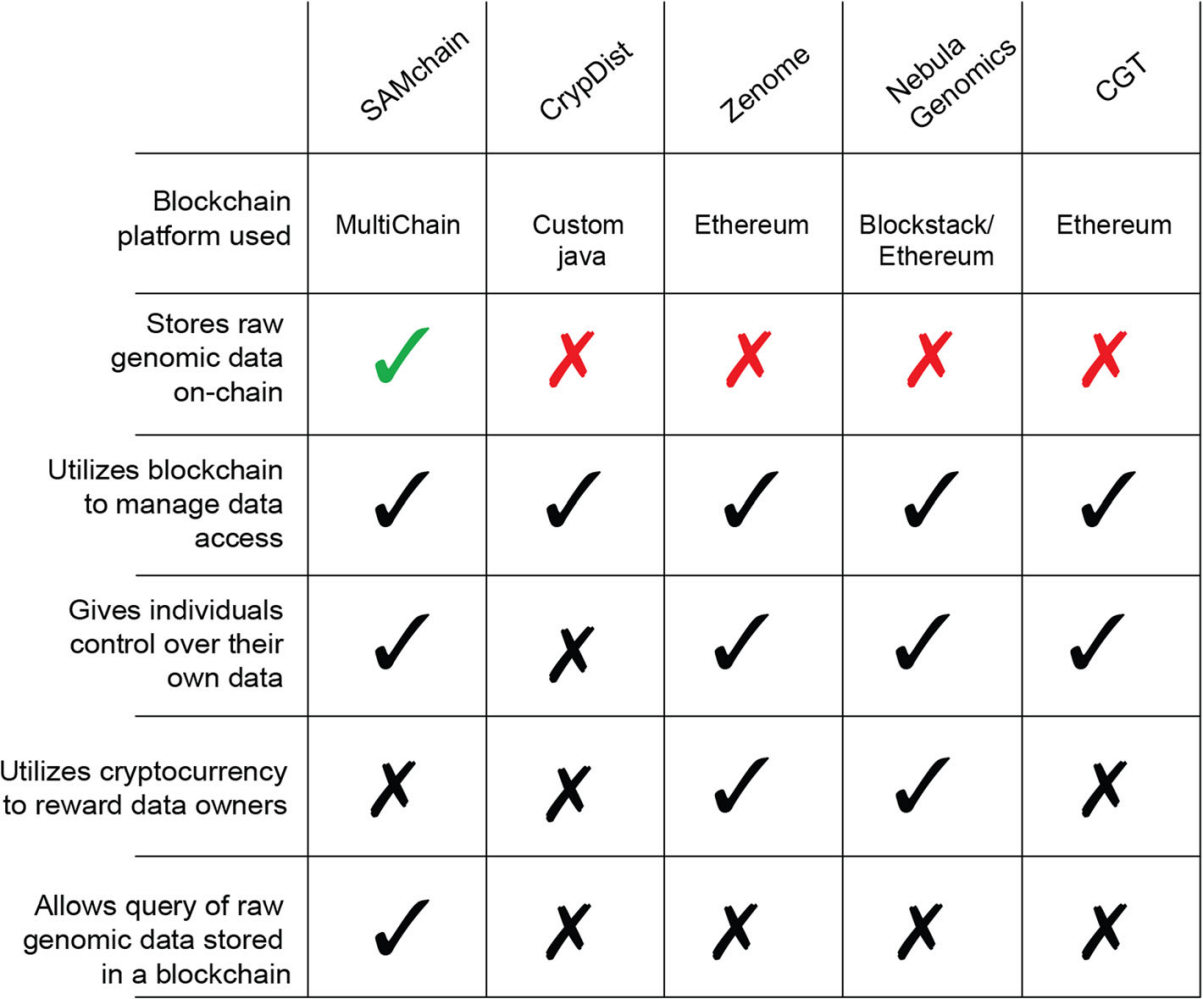
Side-by-side comparison of existing blockchain genomics data storage platforms and SAMchain. Importantly, SAMchain is the only platform that stores raw genomic data on-chain, leading to high-integrity data storage

There are also non-blockchain infrastructures to store raw genomic data in a distributed and secure way. For example, IPFS is a distributed peer-to-peer network. Users in the network can store a file by its content address, and other peers in the network can find and request the file from any node using the distributed hash table implemented in IPFS. Although blockchain and IPFS are based on similar decentralization concepts, IPFS is a peer-to-peer file sharing system. By contrast, our SAMchain and SCTools are designed for the purpose of securely accessing and analyzing the data in a granular fashion, allowing users to index NGS data at the base-pair resolution. IPFS allows hashing the data at the file level and one would have to download the entire SAM file to be able to analyze and query base-pair resolution information. Another technology that allows sharing and analyzing of distributed genomic data is Arvados, which is an open-source platform for large scientific and biomedical data. It allows spinning of cloud computing instances using data that are decentralized to avoid expensive data transfers. It is a bioinformatics platform, in which an application such as SAMchain and SCTools can be implemented and run by instantiating cloud computing nodes. However, it is designed for the use of scientists requiring execution of Common Workflow Language-based scripting that might be difficult for clinicians and patients to adopt. Note that IPFS and Arvados, blockchain, and SAMchain/SCtools are on different conceptual layers hence they cannot fully substitute for each other. In particular, while IPFS and Arvados are useful file systems and computing infrastructures that any application can be built on top of, SAMchain and SCtools are applications that are built on top of blockchain (Additional file 1: Fig. S9).

### SAMchain is user friendly and easy to use by users with no blockchain knowledge

The design of SAMChain and SCtools provides several user-friendly options to scientists with no blockchain background. The modules in SAMchain and SCtools are developed in Python and allows for storing and querying of data using a command line. This allows users to combine the functionalities of our tools with commonly used command line bioinformatics tools such as BEDtools (Additional file 1: Fig. S6). Our codebase also alerts users about potential data storage issues: It regularly checks the remaining storage space during the data storage process. If there is not enough space to add the next read to the chain, it seals the chain and returns a storage error with an indication where the chain has failed. With this, users do not have to rebuild the chain but can continue adding to the chain from where it failed after increasing the available storage (Additional file 1: Fig. S7). Furthermore, our system works well in a high-performance computing (HPC) setting. This means, as long as access is granted, scientists can easily install the MultiChain client and SAMChain app and connect to an existing private blockchain network to analyze genomics data by using their institutions’ existing HPC infrastructure.

## Discussion

We envision a real-world scenario in which individuals create private blockchains to store their personal genomes to share with their healthcare providers and biomedical researchers. Simply with intrinsic access control and security properties, healthcare providers and geneticists can stream or query patients’ genomes. For example, every time a piece of data is encrypted in a shared server or cloud, there is a potential for it to be corrupted, especially if the data are extremely large such as genomic data. Moreover, traditional access-control mechanisms such as dbGAP or EGA leave the research participant or patient outside of the decision-making process. Our private blockchain network reduces not only the risk of data corruption, but also non-permissioned access to private data and gives the control to the actual owner of the data. Blockchain provides immutability such that the data cannot be altered, whether intentionally or accidentally. This is an important aspect of the system not only for personal, private data but also for large-scale open genomic data. Large-scale open data such as those from the 1,000 Genomes Project or the Personal Genomes Project, especially in centralized data storage systems, are vulnerable to corruption and tampering. Moreover, our blockchain-based system can also be combined with blockchain-based data access auditing solutions [5, 19] to provide further control and auditing of access in a more efficient way.

Our framework is the first open-source application to allow querying and streaming of genomic data from blockchain to the best of our knowledge. This is a substantial improvement over the current biomedical applications of blockchains. To address privacy concerns, our framework may be extended to store encrypted data in the data streams. For example, homomorphic encryption has recently been applied to many bioinformatics problems [5, 17, 20–22]. It enables direct computations of encrypted data within the public cloud or any other shared server, providing mathematical privacy guarantees. One could even encrypt the data homomorphically, allowing direct computations on the encrypted data. However, this would add further storage and computation overhead.

Please note that, in our study, each private blockchain network corresponds to a single genome owned by the individual to which the genome belongs. In addition, a single genome will always have an approximately fixed size (for example, 30X whole-genome sequencing will always have 400–600 million reads based on the read length). That is, genomes from millions of users translate into millions of private blockchain networks such that each network must scale only to a fixed, single genome size. Therefore, we believe that neither storage nor the time to push the data (one time cost) will be an issue moving forward if this technology is widely adopted. Moreover, researchers can subscribe to multiple networks at once to query data in parallel from multiple genomes. To summarize briefly, we think that the scalability will not be a problem moving forward, because (1) the genome is fixed in size and we showed that it can be stored on the blockchain with the current data storage options we have; (2) the cost of pushing data, although seems high, is a one-time cost and is attainable, as we showed with our parallelized system; (3) it is designed to hold a single human genome; (4) for storing many genomes, we would create many SAMchains operated by the owner of the genomes.

While the main benefit of using blockchain for data storage is data security and integrity, blockchain also makes it easy to append data to large data files. For example, in the case of SAM files, if a user wishes to add to the data, one could create data streams for these additions (since it is a private chain, only the owner and the permissioned users of the chain would be allowed to make these changes). Thus, the data owner does not have to deal with opening, modifying, and re-indexing large data files, which creates costly network traffic. Searches by genomic location could also check the new data streams to determine if the owner has appended any changes to the data. Furthermore, the stream format lends itself well to storing reads; as discrete, independent items, *ie.,* reads naturally fit into stream format. To make the computation completely on-chain, one could adopt SAMchain and SCtools for Ethereum. To demonstrate how this might be accomplished, we provide a sample Smart Contract in our SAMchain code repository. As Ethereum becomes more and more suitable for database development, this will be an interesting future direction. Another future direction is to create dictionaries from the SAM files, in addition to reference-based compression techniques, compatible with blockchain querying mechanisms in order to further reduce chain storage requirements.

There are other technologies such as IPFS and Arvados that allow for the storage, movement, and computation of large-scale genomic data. IPFS hashing is done at the file level compared to the read level indexing provided by SAMChain. Read level indexing allows more granular queries and analysis of the data (such as variant calling) while the data is still on the blockchain. Arvados provides a platform for bioinformatics analysis that allows for access and audit control through recorded logs, data integrity through hash-based identifiers, privacy through encryption of data at rest, and transmission security through encryption of data in transit. Although its security guarantees are not at the level of what a blockchain provides (e.g., immutability of the data), it has high potential to transform data sharing and analysis in a research setting. However, it is designed for use by scientists and therefore can be difficult for patients and clinicians to adopt.

**Fig. 5:**
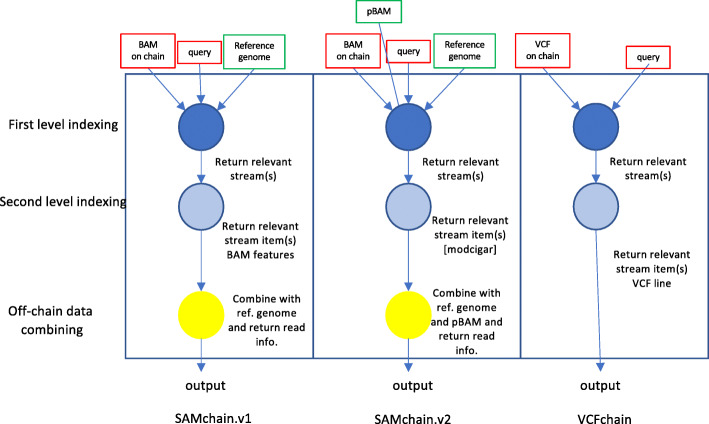
Schematic of storing .diff files in our blockchain implementation and the differences between the various implementations introduced in this study

Our blockchain solution can be generalized to other large-scale data storage and querying problems beyond genome sequencing data. For example, functional genomics assays are generating an unprecedented amount of data from different modalities. Studies have shown that privacy concerns will lead to a large fraction of these data being siloed behind a firewall. Therefore, we recently proposed privacy-preserving BAMs (pBAMs) as an alternative approach to the current controlled-access functional genomics data sharing mechanisms [23]. pBAMs, which have the same structure as SAM/BAM files, allow for the public sharing of read alignments of functional genomics data while protecting sensitive information and minimizing the amount of private data that requires special access and storage. Because genetic variants are often unnecessary for most downstream calculations, the public component (pBAM files) and the private component (.diff files) of the aligned reads are differentiated and only the private files are stored behind a firewall. We envision that one can further optimize the data storage in SAMchain by storing only the .diff component on-chain. That is, only the private information (i.e., the genetic variants) per read could be stored on-chain, while the rest of the information of the reads (i.e., the pBAM files) could be stored off-chain (Fig. 5). Data including but not limited to functional genomics data, VCF files from multiple individuals, and somatic mutation datasets from cancer patients can be stored in blockchain using our indexing schemes, allowing for rapid and partial retrieval of the data. Note that our system is based on the existing reference genome-based file formats. However, we envision that these file formats might be modified or replaced by other formats that use graph genomes and pan-genomes as references [24–26]. This will likely change the indexing and querying mechanisms that we propose in this study.

## Conclusions

Blockchain is an exciting new technology that has provided solutions to ownership and integrity challenges in other realms (e.g., finance and art). However, its use in genomics is stymied by the fact that storing large-scale data on a blockchain can be challenging. Here, we provide a solution to overcome these roadblocks. We developed a novel private blockchain network to store personal genomic variants and reference-aligned next-generation sequencing reads on-chain using nested database indexing. We also developed tools for rapidly accessing and analyzing the on-chain data. We addressed the challenges of on-chain data storage by minimizing the data inserted to the chain using reference-based data compression and indexing techniques. Moreover, our tools provide open-source blockchain-based storage and access for advanced genomic analyses such as variant calling.

## Methods

### MultiChain API

We designed SAMchain as a layer on top of MultiChain (Additional file 1: Fig. S8). MultiChain data streams make it possible for a blockchain to be used as a general purpose database. The data published in every stream is stored by all nodes in the network. Each data stream on a MultiChain blockchain consists of a list of items. Each item in the stream contains the following information, as a JSON object: A publisher (string), key:value pairs (from 1 to 256 ASCII characters, excluding whitespace and single/double quotes) (string), data (hex string), a transaction ID (string), blocktime (integer), and confirmations (integer). When data need to be queried or streamed, they can be retrieved by searches using the key:value pairs. Publishing a stream item to a data stream constitutes a transaction. When a node subscribes to a stream, it indexes the stream items in different ways to enable fast retrieval, and the index entry points to the transaction ID. Because of the peer-to-peer network architecture, stream items can arrive at different nodes in the network in different orders. For more details please see MultiChain whitepaper at https://www.multichain.com/download/MultiChain-White-Paper.pdf (referred as Greenspan 2015 throughout methods section [last access June, 2022]).

### MultiChain in a blockchain network

MultiChain permits the user to set a network ordering parameter to be local or global (the default is global, used for SAMchain). Global ordering means that once the chain has reached consensus, all nodes see the same order in their streams. Transactions submitted to the network are time stamped via the Linux timestamp. When a transaction occurs, it is held in the memory pool. After mining of the transaction is complete, the transaction is added to a block. Each block has a maximum transaction size, i.e., after a block reaches its maximum size or the time to create a block reaches its limit, the block is sealed and appended to the chain. This means that a data stream in MultiChain can span multiple blocks based on the time of the transaction (i.e., the time of publishing the data to the blockchain). New blocks are created according to the “target-block-time”, a parameter set upon initializing a chain.

### Multichain and blockchain mining

For "target-block-time", we used the default value, which is 15 s. We also used the default consensus mechanism, which is a round-robin schedule of miners (Greenspan 2015). Users can also turn on proof-of-work mining if they desire, but the security of MultiChain does not depend on the proof-of-work scheme. As outlined in the MultiChain whitepaper (Greenspan 2015, pg. 3), problems can arise if proof-of-work mining (used in the public Bitcoin blockchain, for example) is applied to a private or “institutional” setting, such as the “51% attack,” in which over half of the permissioned participants collude to alter the chain. To resolve this issue, MultiChain makes use of a “mining diversity” parameter, which controls the number of blocks that may be created by a given user within a set time window. Tuning this parameter changes the proportion of the network that would need to collude in order to undermine the network (detailed on pg. 7–8 of Greenspan 2015). Therefore, even though MultiChain is a private network, immutability is achieved.

We designed SAMchain, SCtools, VCFchain, and VCFquery using MultiChain version 2.0.3 and Python version 2.7.16. Together, the SAMchain and SCtools repositories contain six modules: buildChain, insertData, buildBAM, queryReads, queryDepth, and pileup. VCFchain and VCFquery contain three modules: buildChain, queryPosition, and queryGT-RSID.

### SAMchain design overview

We took an approach to maximize the efficiency of storing and querying data. Our goal was to store minimal data while indexing it in a creative way to allow rapid retrieval, thereby reducing the time and memory cost of analysis and increasing the utility of the stored data (Fig. 1a). To achieve this goal, we manipulated (a) data structures in data streams and (b) data to be stored in SAM files. For (a), we first separated mapped and unmapped reads from a SAM file. First, we created a data stream called metaData to store the header data and general information (bin size, number of streams, etc.) about the chain. We then created N streams (called chr_i-bin_j). Each of these N streams represents a bin of genomic coordinates. Based on the location of a read mapped on the human reference genome, we logged the read names as data in chr_i-bin_j stream. Some reads span two bins. In that case, we stored the read in the bin to which the beginning of the read maps. We then added a boolean key to the chr_i-bin_j stream that we call FLANK (Additional file 1: Fig. S1). FLANK = 0 indicates that the entire read is in that bin (Additional file 1: Fig. S1). FLANK = 1 indicates that the read coordinates span two consecutive streams. The FLANK value tells our retrieval algorithm to search for a particular read in two chr_i-bin_j streams. Our query algorithm can retrieve the data in the chr_i-bin_j stream based on the queried location. Our code base allows developers to bin the data according to a desired feature that might be queried by users such as read names, mapping qualities, or alignment scores; these are the features stored as keys in the binned streams. Our implementation uses binning by genomic location, as it is the most commonly queried property for depth analysis or variant calling. Unmapped reads are stored in a separate stream called unmappedANDcontigs, but not in the chr_i-bin_j streams. For (b), we were inspired by the data compression techniques in CRAM files (Hsi-Yang Fritz et al. 2011, Gursoy et al. 2019) and stored the difference between the read and the reference sequence in the chain instead of the sequences themselves to reduce the size of the data stored on-chain With this approach, our implementation is able to regenerate the sequence of a read by using the reference genome and other features stored in the chain.

### SCtools design overview

We developed SCtools to extract information from SAMchain for downstream analysis. We provide a code base that has the ability to query on a blockchain. The key:value property of the data streams in MultiChain2.0 together with the ability to query on multiple keys provides an opportunity to extract data from the blockchain without the need for costly calculations. Our query module can retrieve data from a chain based on the position in the reference genome (Fig. 1c).

If a user queries the chain for reads mapped to a genomic region, our query module first finds the correct streams/bins containing that region. From the bins, it extracts the SAM data and MODCIGAR and uses an input reference file to return the results. This approach reduces the query time significantly for the following reason: Data streams do not allow range searches. If the data were kept in a single stream, then the query would have to iterate over the location range for every single stream item. With binned streams, the query is only done on the streams containing the relevant data.

Below, we describe the functionality of each SAMchain, SCtools, VCFchain, and VCFtools module.

#### buildChain (owner node)

buildChain initializes a MultiChain blockchain and creates streams that define the SAMchain. Three types of streams exist in a SAMchain: (1) metaData, (2) unmappedANDcontigs, and (3) binned streams. metaData is a single stream that stores SAMchain settings (bin length, read length, and number of bins) and eventually stores the header from an input SAM file. unmappedANDcontigs is a single stream that stores the features from the input SAM file, except for the sequence and quality string, for unmapped reads and contigs. When parsing an input SAM file, the insertData module uses the FLAG feature to determine whether to put a read into the unmappedANDcontigs stream or a binned stream. Binned streams are a series of streams that map to a range of positions in the genome. buildChain divides each chromosome into *N* kb intervals (*N* is set by the developer) and creates a stream for each interval. We made this design choice to improve query efficiency. A user’s query leads to a specific binned stream (or set of streams), rather than to all the data. See Additional file 1: Fig. S2 for the flowchart of this module.

#### insertData (sequencer node)

insertData pushes data from an input SAM file to the relevant streams in an initialized SAMchain. First, insertData uses PySAM to extract the header data from an input SAM file and pushes the header, line-by-line, to the metaData stream. Next, it uses PySAM to extract the features of the reads in the SAM file, one at a time, and checks the read’s flag feature to determine whether it belongs in the unmappedANDcontigs stream, or to a binned stream. It then pushes the read features to the appropriate stream as the data field of a single stream item. It checks whether the read’s position spans two streams. If it does, it stores that read in the stream mapping to its start position and stores “FLANK = 1” as a key. If it does not, it stores “FLANK = 0” as a key.

#### queryReads (clinician/researcher node)

queryReads searches a SAMchain for reads that match an input region of interest in the genome. It first pulls information from the metaData stream about how the reads were binned during buildChain and uses it to obtain the names of the stream(s) that correspond to the input genomic location. If the first stream in this list is not the first stream of a chromosome, it adds the stream name just upstream, in the case that a FLANK = 1 read is present in that stream. Given these stream names, it uses the built-in MultiChain commands liststreamitems and liststreamkeyitems to retrieve the items from those streams and check whether they match the region queried. Then, using the modcigar, it extracts the correct sequence from the reference genome and returns the results. See Additional file 1: Fig. S3 for the flowchart of this module.

#### queryDepth (clinician/researcher node)

SCtools provides a useful function to determine the sequencing depth for a queried location or all of the locations in the genome. queryDepth follows a similar algorithm as queryReads. However, after obtaining the read data, queryDepth must check the cigar values for each read in order to calculate depth, taking into account information about insertions and deletions (for example, if a deletion occurs at a location queried in one of the reads, this should contribute + 0 to the depth at that location). After calculating the depth values, queryDepth returns the results. See Additional file 1: Fig. S3 for the flowchart of this module.

#### pileup (clinician/researcher node)

SCtools provides a useful function to determine the pile-ups for a queried location or all of the locations in the genome. Pile-up files contain the number of reads that mapped to a location, the reference allele for that location, and the sequenced nucleotide in each read for that location. This allows users to visualize the genetic variation and calculate allele frequencies for the variants. pileup follows a similar algorithm as queryReads. However, after obtaining the read data, pileup must check the cigar values for each read in order to output pileup, taking into account information about insertions and deletions. After doing so, pileup returns the results. See Additional file 1: Fig. S3 for the flowchart of this module.

#### buildBAM (clinician/researcher node)

buildBAM rebuilds a BAM file from the data stored in a SAMchain. It first retrieves the header data from the metaData stream. Next, it retrieves the data from the binned streams and converts it to a tab-separated format. Using PySAM, it extracts from an input reference file the sequence string and alters it based on the cigar. Finally, it uses PySAM to write the read entry to an output BAM file. See Additional file 1: Fig. S4 for the flowchart of this module.

Additional file 1: Fig. S5 summarizes the overall structure of the SAMchain and Sctools.

### VCFchain and VCFquery

#### buildChain

buildChain initializes a MultiChain blockchain and creates streams that define the VCFchain. Two streams exist in VCFchain: (1) metaData and (2) allVariantData. metaData eventually stores the header from an input SAM file. allVariantData stores the features from the input VCF file, with genomic position, genotype, and rsID as the keys. All variants are inserted to the allVariantData stream.

#### queryAND

Because positions in a VCF file are unique, queryAND retrieves VCF feature entries from the allVariantData stream using input genomic position as the key. It can filter data based on an input genotype and/or variant ID.

## Supplementary Information

**Additional file 1: Supplementary Information.** This files includes all the supplementary text, figures and tables cited in the main text.

**Additional file 2.** Review History.

## Data Availability

SAMChain and SCTools can be found at https://github.com/gersteinlab/SAMChain and DOI:10.5281/zenodo.6573999 under MIT Licence [27]. The Ethereum smart contract and VCFChain code can also be found at the github page. The SAM file (BAM) used in this manuscript can be found at http://files.gersteinlab.org/public-docs/2022/02.23/HG00114.loci.bam. The VCF file used can be accessed at https://www.encodeproject.org/files/ENCFF907ASL/ [28].
