# Supplementary material for: Storing and analyzing a genome on a blockchain
Source: Genome Biol. 2022 Jun 29;23:134. doi: 10.1186/s13059-022-02699-7 (PMC9241283; doi:10.1186/s13059-022-02699-7)
Supplement: Supplementary file 1 — Additional file 1: Supplementary Information. This files includes all the supplementary text, figures and tables cited in the main text. [file 13059_2022_2699_MOESM1_ESM.pdf]

Supplemental Information

A blockchain primer

In recent years, Blockchain has emerged as a potential solution that would achieve these requirements. Blockchain has several key properties which could make it an ideal solution, including a decentralized, distributed architecture, and cryptographic protocols that yield immutability. Already today, there are multiple personalized-medicine startups that aim to use blockchain to improve genomic data storage.

Proposed in 2008 as a global cryptocurrency network (Nakamoto 2008), blockchain is now used to solve a variety of problems. Blockchain consists of an append-only data structure shared in a decentralized, distributed network, which can be public or private. The network relies on a consensus mechanism to add to the chain. Most widely known is proof-of-work (PoW), in which nodes in the network participate in a mining competition. Others are proof-of-stake (PoS) and proof-of-authority (PoA). Public blockchain networks typically make use of PoW, which is suitable for large networks of individuals of unknown identity and intent. On the other hand, private blockchain networks consisting of individuals with known, semi-trusted identity tend to use PoA. A previous study highlights the specific benefits of blockchain for biomedical research applications (Kuo et al. 2017). These include decentralized management, immutable audit trail creation, data provenance, robustness and availability, security and privacy.

There are a few different blockchain platforms one might consider for developing a data-storage application. One is Bitcoin, a public cryptocurrency network. However, here we are concerned with private blockchain networks, as genomic data is sensitive and should only be shared with a set of individuals or institutions (e.g. sequencing centers, physicians, biomedical researchers) (Figure 1a). Whereas anyone in the world can participate in the public Bitcoin blockchain, only permissioned individuals can sync a private blockchain. Furthermore, Bitcoin supports only simple transactions and transfers of small amounts of data (~80 bytes) from one user to another. Another is Ethereum, which supports more complex transactions via Smart Contracts, self-executable on-chain programs which write to their own storage. Ethereum is a public network, but permits the creation of private networks. One of the most prominent platforms for private-blockchain development is MultiChain, a Bitcoin-like platform. Different kinds of blockchains are suitable for different use cases. For example, the Bitcoin blockchain is perfectly suitable for cryptocurrency exchanges. It simply acts as a ledger of transactions between different accounts. However, for more complex transactions or storage protocols, Ethereum becomes more suitable. MultiChain does not permit on-chain computation as Ethereum does, but it has several features that make it especially suitable for data storage use cases. Both the MultiChain platform and Ethereum Smart Contracts have been previously used in medical genomics applications, but in limited settings (Gursoy et al. 2020; Pattengale and Hudson 2020; Ozdayi et al. 2020; Ma et al. 2020; Gursoy and Brannon et al. 2020).

Previous reviews outline the current status of commercial and academic proposals that use blockchain technology to improve genomic data sharing (Ozercan et al. 2018; DeFrancesco and Klevecz et al. 2019). Among these platforms are CrypDist, Zenome, Nebula Genomics, the Cancer Gene Trust, and Encrypgen/Gene-Chain. Each of these platforms utilizes blockchain for different aspects of the genomics data storage and sharing process. For example, Zenome makes use of Ethereum Smart Contracts to facilitate access to genomic data files and exchange of 'ZNA tokens', cryptocurrency which allow individuals to be compensated for their genomic data (Ozercan et al. 2018; Kulemin et al. 2017; Zenome.io 2017). Nebula Genomics also uses Smart Contracts to communicate between nodes in the network, survey participants, and facilitate data access permissions (Grishin et al. 2018). However, due to the difficulty of storing large data on blockchains, many of these companies store the genomic data elsewhere, such as in Blockstack or InterPlanetary File System (Grishin et al. 2018; Currie 2018). For example, CrypDist uses a custom Blockchain to store *links* to genomic data files (such as reference-aligned BAM files), which are actually stored in AWS data buckets (Sahin 2017). Because these platforms do not actually store genomic data 'on-chain', they are missing a key benefit of blockchain: high-integrity, secure data storage. Storing links to data in a blockchain can be useful in some cases, for example, if it is important to keep an access log for a particular dataset (Gursoy et al. 2020). Yet, it does not secure the data itself, nor does it prevent it from being altered, as it is stored somewhere else entirely. On the other hand, storing genomic data on-chain maintains the integrity and security of the data (Kuo et al. 2017) . Additionally, while many existing platforms provide network architectures for storing genomic data, few offer solutions for performing computation on the data stored in the network. This is a critical gap in the technology; not only do clinicians and researchers need access to high-integrity, raw genomic data, but they also require secure tools for querying and streaming the data. Moreover, computation and querying data directly from blockchain allow data owners to give clinicians and researchers partial access to the data, while existing platforms follow a "all or no access" model. This is likely due to a central caveat of blockchain technology: the inefficiency of storing and querying data due to the potential for chains to reach large sizes. The storage space and computational power required by blockchain is greater than a centralized database application due to the redundancy of storage and network verification protocols. The decentralized system also creates a higher latency (delay in data communication) during storage and retrieval of data. Additionally, transactions in the blockchain network require a cryptographic consensus verification, which makes them slow to publish data to the chain (Nakamoto 2008).

Details of other blockchain platforms:

CrypDist: CrypDist is a program initially developed as an undergraduate senior project (Ozerkan et al. 2018). The code base for CrypDist is written in java and available on Github (Sahin et al. 2017). CrypDist does not make use of any widely used blockchain platform (such as Ethereum or MultiChain), but rather constructs a custom blockchain in java. The CrypDist blockchain stores links to data files, which appear to be stored in AWS buckets (Sahin et al. 2017). Importantly, the role of blockchain here is to store links and to log access to data rather

than the data itself. This application is useful for research purposes when there are multiple NGS files from a cohort of individuals, as storing all the alignment files from the cohort in a blockchain is not feasible due to the storage requirements. This application aims to protect the integrity of metadata, while the security and integrity of the actual SAM files are not provided by blockchain. SAMchain differs from this application because 1) it stores data on-chain, 2) it gives individuals control over their data, 3) it is better suited for a personal genomics use case, that is, individual-level analysis rather than population-level analysis.

Zenome: Zenome is a company founded in 2017 (Kulemin et al. 2017; Zenome.io 2017). It appears that Zenome uses Ethereum Smart Contracts to facilitate ownership of and compensation for data (Zenome.io 2017). Nodes in the network can sell their data or services for “ZNA tokens,” cryptocurrency convertible to ether (Ethereum currency). Raw genomic data is not stored in the Smart Contract. According to our understanding of their whitepaper and Smart Contract code, it appears that the data is stored off-chain in a distributed file storage system (Zenome.io 2017; Ozercan et al. 2018). The main differences between SAMchain and Zenome are 1) SAMchain stores NGS data on-chain to maximize data integrity, and 2) SAMchain does not utilize cryptocurrency.

Nebula Genomics: Nebula Genomics is a personal genomics company founded in 2018 (Grishin et al. 2018). According to their whitepaper, the Nebula system partitions genomes into overlapping, variable-length sequences and represents each tile as a hash digest of the sequence it contains (Grishin et al. 2018). Data storage and access control is implemented using Blockstack. Blockstack makes use of blockchain, but not for storage; files are stored in a local drive or in the cloud (Digital Ocean, S3, Dropbox). The tile library of individual genomes (referenced by hash arrays) is stored in public storage such as InterPlanetary File System. Ethereum Smart Contracts are used to communicate among nodes, survey participants, and purchase access to data, but not to store data. (Grishin et al. 2018; Blockstack docs; Defrancesco and Klevecz 2019). As in the case with Zenome, Nebula uses blockchain to keep track of transactions and provide incentive for individuals to share their data. On the other hand, SAMchain uses blockchain to store raw genomics data on-chain.

Cancer Gene Trust: The Cancer Gene Trust (CGT) was built in 2019 (Defrancesco and Klevecz 2019), and is currently being run as a pilot program through UCSF. Their documentation describes an “a lightweight, global off-blockchain decentralized network controlled by "stewards" that make limited somatic mutation and related clinical data about a patient publicly available.” (Currie 2018). The data is stored off-chain in InterPlanetary File System, a peer-to-peer distributed file sharing system. Raw sequencing data is stored locally by data stewards. The blockchain component of CGT is Ethereum–a smart contract stores references to data files in the form a multihash of their content. Importantly, genomic data itself is not stored in Ethereum (references to the data are), and raw genomic data is not made available to the public CGT. Rather, blockchain is used as a tool to facilitate access to distributed files similar to CryptDist.(Cancer Gene Trust 2018; Currie 2018; Defrancesco and

Klevecz 2019; Ozercan et al. 2018). SAMchain differs from CGT in that SAMchain stores NGS data on-chain to maximize data integrity.

Encrypgen/Gene-Chain: Encrypgen is a company founded in 2016 (Picco 2019) which has released Gene-Chain. Gene-Chain is a self-described DNA data marketplace. Their primary market is current users of 23andMe (Gonzalez and Kopsell 2020). Gene-Chain allows data owners to receive monetary compensation for access to their data via DNA tokens, though exactly how this works is not fully explained (Ozercan et al. 2018). From the available code, we can tell that the blockchain platform used is MultiChain. However, the code published here appears to be the multichain-web-demo repository (published by multichain), rather than the code showing how Gene-Chain works. Their use of MultiChain is also confirmed in their white paper (EncrypGen 2018). However, this white paper does not include any description of technical specifications. It is not clear whether the data will be stored in a MultiChain stream, or elsewhere. (Ozercan et al. 2018; encrypgen 2017; EncrypGen 2018; Defrancesco and Klevecz 2019). Due to the limited information available, we are not able to make a fair comparison between our application and Gene-Chain. However, we can tell that one key difference is the focus of the application. While Gene-Chain focuses on compensation and transactions, SAMchain focuses on high-integrity data storage and efficient querying.

In summary, most of the existing biomedical blockchain applications use blockchain for its cryptocurrency & transaction properties, while SAMchain and SCtools utilize it for data integrity purposes. In Table 2, we provide a concise comparison of these four blockchain platforms along with SAMchain.

Storing metadata vs. the data itself on the blockchain:
Here we present a simple analogy to illustrate why storing metadata and links to genomic data does not achieve data security. We can think of genomic data as books in a library. We can log the location (i.e. aisle) of each book in the library using a secure software. However, the books themselves will still be physically kept in the library, a single location. If there were a fire in the library, the logged information would not be affected, but we would still lose all of the books. In the same way, if we kept only the links to genomic data in a blockchain and the data itself elsewhere in a centralized storage system, the data would be lost if the centralized storage system were to fail. In other words, the security properties of blockchain would not be beneficial to the data itself.

MultiChain provides the most advantages to the SAMchain use case

We first evaluated the advantages of major blockchain platforms, Bitcoin, Ethereum, and MultiChain, for our particular use case (Table S3):

- Bitcoin is primarily designed to be a public cryptocurrency blockchain network, and does not offer an efficient way of indexing and querying stored data. Thus, we quickly ruled it out for our particular use case.

- Ethereum makes use of Smart Contracts, which are Turing-complete, on-chain programs that can perform many different functions (other than just sending value from one account to another). While Ethereum can be used as a distributed database, it is more suitable for scientific computation.
- MultiChain is perhaps more suitable for database development due to built-in features called "streams". Streams are append-only, on-chain lists of data with key:value retrieval capability, making store and query functionality extremely easy. From a developer's perspective, MultiChain also has advantages of centralized documentation and ease of implementation.

Given that we wanted to develop a robust software usable by individuals, physicians, and researchers for storage, query, and computation, we developed SAMchain with MultiChain. While MultiChain does not currently allow for on-chain computation like Ethereum does, its natural capacity to be used as a database outweighed this disadvantage. We provide an Ethereum Smart Contract in our code base as an example of how one might store raw genomic data in a Smart Contract, which would permit on-chain computations on the genomic data. As Ethereum becomes more widely utilized for database use cases, SAMchain could be expanded to Ethereum. In SI Table 1, we summarize the big-picture differences between Ethereum and MultiChain.

MultiChain storage in a multi-node network

On a given node, MultiChain keeps a "wallet" directory on the disk, in addition to the blocks, which stores transactions of particular relevance to that node (i.e. transactions initiated by that node). This protocol is inherited from Bitcoin, however Bitcoin holds these transactions in memory while MultiChain stores them on disk (MultiChain 2020: Announcing the new MultiChain wallet). For example, let us say that a MultiChain network consists of three nodes A, B, and C, each node syncing the chain. If any of the three nodes publishes data to a stream, that data will be embedded in a block stored on each node. However, if node A is the one to publish all the data, node A will use more storage than B and C because in addition to storing the blocks, node A will also keep these transactions in its wallet. In the SAMchain network ecosystem, the vast majority of transactions happen at the initialization of the chain when the SAM data is published by the sequencer node. Thus, in Figure 2 Panel b, the first node (which published the SAM data to the chain) requires ~23 GB for this particular test dataset, while nodes 2-4 each require ~7.5 GB to sync the blocks.

Description of SAMChain, SCtools, VCFChain, and VCFquery modules

SAMChain and SCtools

buildChain (owner node): buildChain initializes a MultiChain blockchain and creates streams that will define the SAMchain. Three types of streams exist in SAMchain: 1) metaData, 2)unmappedANDcontigs, and 3) binned streams. metaData is a single stream which stores SAMchain settings (bin length, read length, and number of bins) and will eventually store the header from an input SAM file. unmappedANDcontigs is a single stream which stores the

features from the input SAM file, except for the sequence and quality string, for unmapped reads and contigs. When parsing an input SAM file, the insertData module will use the FLAG feature to determine whether to put a read into the unmappedANDcontigs stream or a binned stream. Binned streams are a series of streams which map to a range of positions in the genome. buildChain divides each chromosome into N kb intervals (N is set by the developer) and creates a stream for each interval. We made this design choice to improve query efficiency. A user's query leads to a specific binned stream (or set of streams), rather to all the data.

insertData (sequencer node): insertData pushes data from an input SAM file to the relevant streams in an initialized SAMchain. First, insertData uses pysam to extract the header data from an input SAM file and pushes the header, line-by-line, to the metaData stream. Next, it uses pysam to extract the features of the reads in the SAM file, one at a time, and checks the read's flag feature to determine whether it belongs in the unmappedANDcontigs stream, or a binned stream. It then pushes the read features to the appropriate stream as the data field of a single stream item. It checks whether the read's position spans two streams. If it does, it stores that read in the stream mapping to its start position and stores "FLANK=1" as a key. If it does not, it stores "FLANK=0" as a key.

***buildBAM (clinician/researcher node)*:** buildBAM rebuilds a BAM file from the data stored in a SAMchain. It first retrieves the header data from the metaData stream. Next, it retrieves the data from the binned streams and converts it to a tab-separated format. Using pysam, it extracts from an input reference file the sequence string and alters it based on the cigar. Finally, it uses pysam to write the read entry to an output BAM file.

queryReads (clinician/researcher node): queryReads searches a SAMchain for reads that match an input region of interest in the genome. It first pulls information from the metaData stream about how the reads were binned during buildChain, and uses it to obtain the names of the stream(s) that correspond to the input genomic location. If the first stream in this list is not the first stream of a chromosome, it adds the stream name just upstream, in the case that a FLANK=1 read is present in that stream. Given these stream names, it uses built-in MultiChain commands liststreamitems and liststreamkeyitems to retrieve the items from those streams and check whether they match the region queried. Then, using the modcigar, it extracts the correct sequence from the reference genome and returns the results.

queryDepth (clinician/researcher node): SAMtools provides a useful function to determine the sequencing depth for a queried location or all of the locations in the genome. queryDepth follows a similar algorithm to queryReads. However, after obtaining the read data, queryDepth must check the cigar values for each read in order to calculate depth taking into account information about insertions and deletions (for example, if a deletion occurs at a location queried in one of the reads, this should contribute +0 to the depth at that location). After calculating the depth values, queryDepth returns the results.

pileup (clinician/researcher node): SAMtools provides a useful function to determine the pile-ups for a queried location or all of the locations in the genome. Pile-up files contain the number of reads that mapped to a location, the reference allele for that location, and the sequenced nucleotide in each read for that location. This allows users to visualize the genetic variation and calculate allele frequencies for the variants. pileup follows a similar algorithm to queryReads. However, after obtaining the read data, pileup must check the cigar values for each read in order to output pileup taking into account information about insertions and deletions. After doing so, pileup returns the results.

VCFChain and VCFtools modules

buildChain: buildChain initializes a MultiChain blockchain and creates streams that will define the VCFchain. Two streams exist in VCFchain: 1) metaData and 2) allVariantData. metaData will eventually store the header from an input SAM file. allVariantData will store the features from the input VCF file, with genomic position, genotype, and rsid as the keys. All variants will be inserted to the allVariantData stream.

queryAND: Because positions in a VCF file are unique, queryAND retrieves VCF feature entries from the allVariantData stream using input genomic position as the key. It can filter data based on an input genotype and/or variant ID.

Compatibility with other NGS Software

We developed SAMchain using Python. We added several wrappers around MultiChain API such that users do not have to know the underlying blockchain technology. SAMchain and SCtools functionalities are used by invoking Python scripts in the command line. In addition to BEDTOOLS, any other software (e.g., BCFtools) or programming language (e.g., AWK) can be piped into SAMChain. SI Figure 6 is an example of how SAMchain might be used with BEDtools.

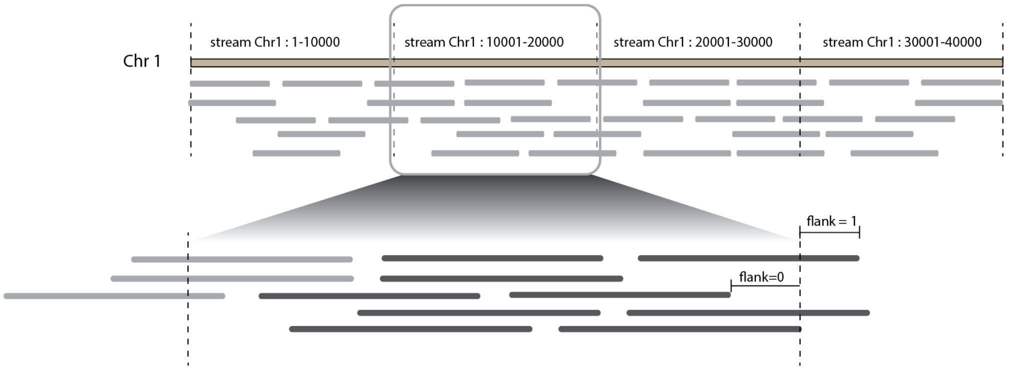

Fig S1. Binning reads by position in streams. To store read data from a sequence alignment file, we define several streams, each with a fixed position window. Some reads may span two of these streams. Thus, for each read we store a field called FLANK, which has value 0 if the data is in one stream, and value 1 if it spans two streams.

	Key	Data
metaData	“chainInfo”	{<br>"json":{<br>"binLength":<binLength>,<br>"readLength":<readLength>,<br>"numBins":<numberBins><br>}<br>}
	“Header”	{<br>"json":{<br>"header":<headerText>,<br>}<br>}
chr{i}stream{j}	“FLANK={1 if hangs, 0 if not}”	{<br>"json":{<br>"QNAME":<readName>,<br>"FLAG":<flag>,<br>"RNAME":<chromosome>,<br>"POS":<position>,<br>"MAPQ":<mapq>,<br>"CIGAR":<cigar>,<br>"RNEXT":<next reference id>,<br>"PNEXT":<next reference start>,<br>"TLEN":<template length>,<br>"SEQ":<query sequence>,<br>"QUAL":<query qualities>,<br>"OP":<tuple of optional tags>,<br>}<br>}

Table S1. Distribution of data across key and data fields of different types of streams.

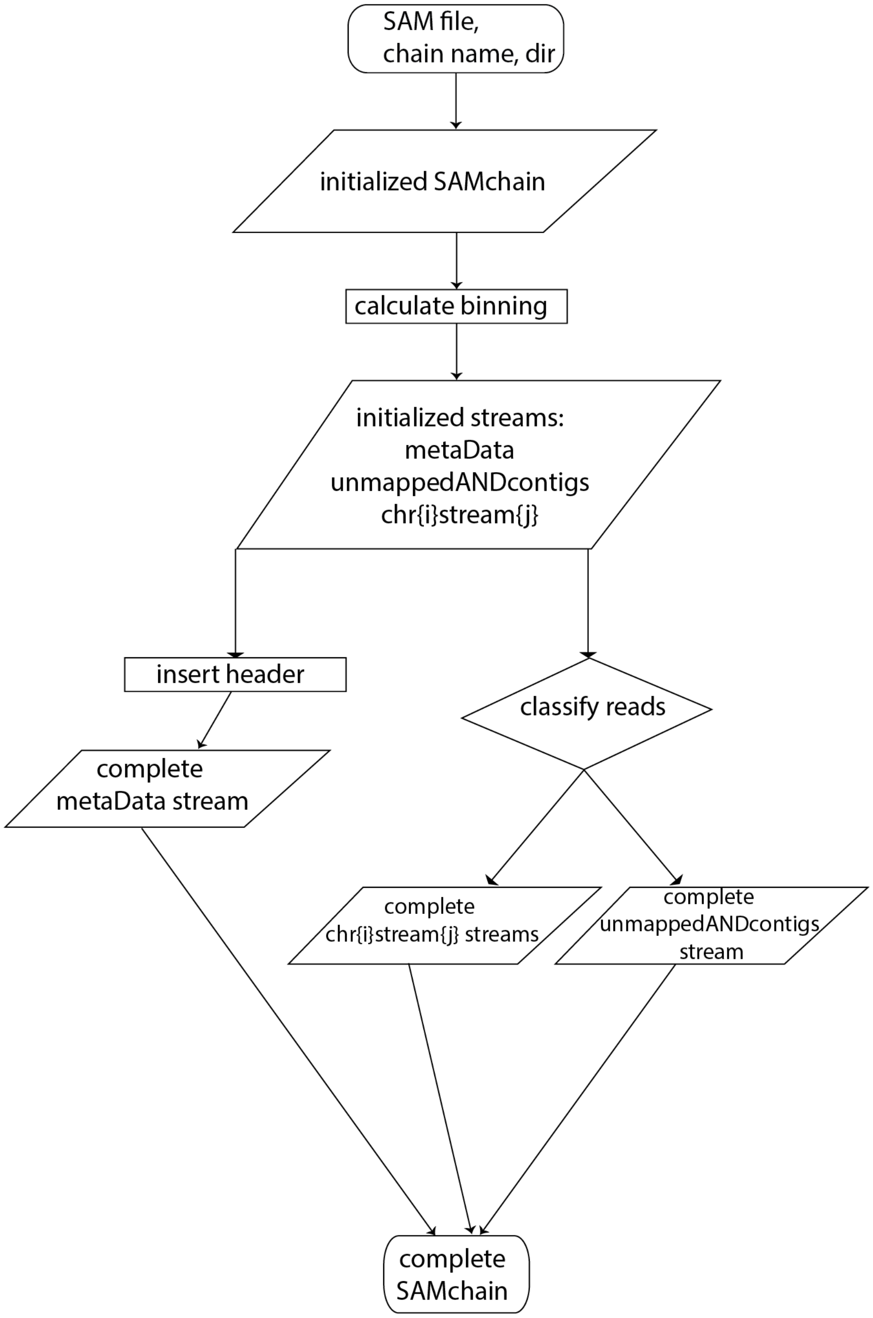

Fig S2. Flowchart for buildChain.py, a module which builds a SAMchain from an input BAM file.

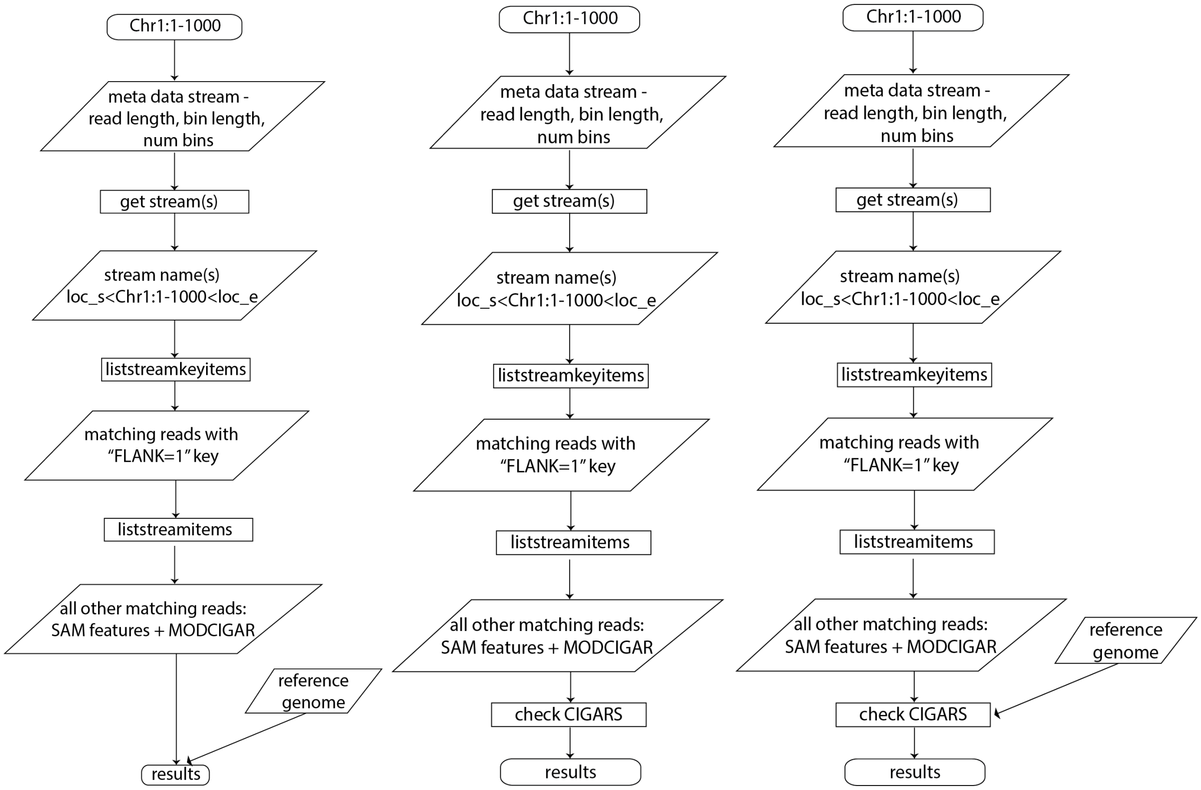

Fig S3. Flowcharts for queryReads.py (left), queryDepth.py (middle), and pileup.py (right), the core modules of SCtools.

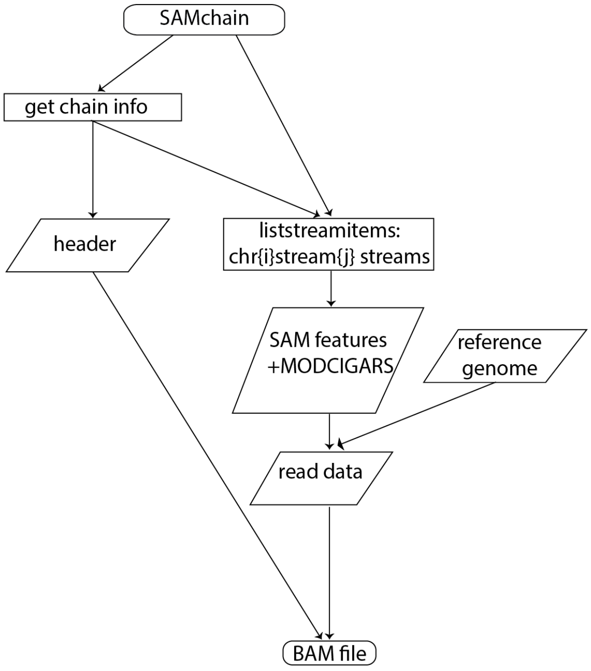

Fig S4. Flowchart for buildBAM.py, a module which rebuilds a BAM file from data stored in a SAMchain.

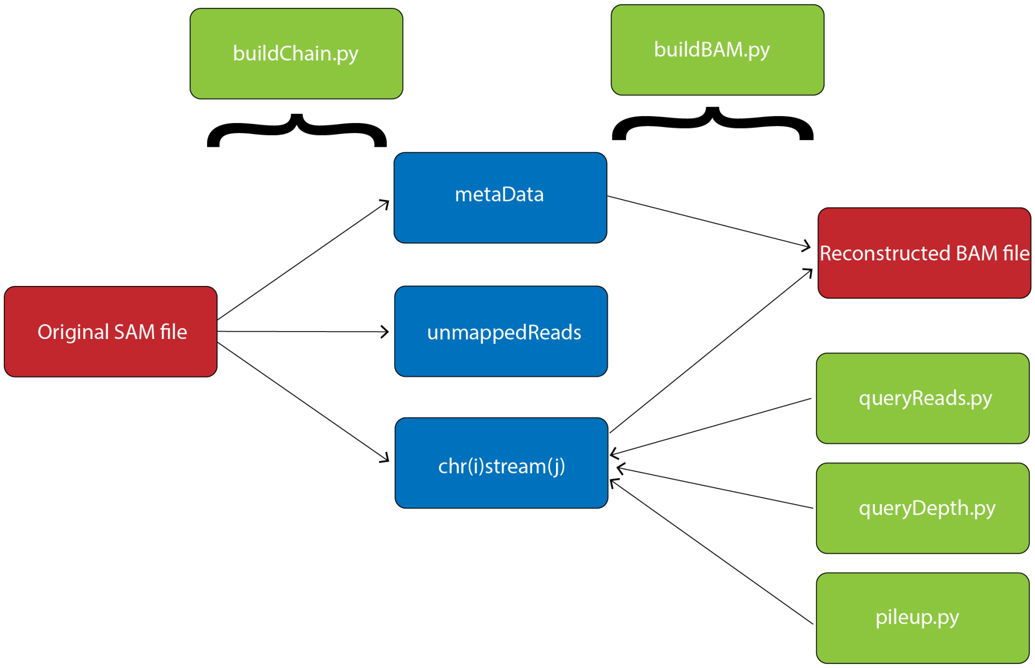

Fig S5. Overview of the SAMchain and SCtools organization and functions.

a)

```
(py2) ak2020@kubeminion1:~/SAMChainProject$ bedtools intersect -a example.bed -b example2.bed          chr1
  56994717        56994817        .       1000    .       1633.58610      -1.00000        4.59542 606
(py2) ak2020@kubeminion1:~/SAMChainProject$ start=$(bedtools intersect -a example.bed -b example2.bed | cut -f2 -d$'\
t')
(py2) ak2020@kubeminion1:~/SAMChainProject$ end=$(bedtools intersect -a example.bed -b example2.bed | cut -f3 -d$'\t'
)
(py2) ak2020@kubeminion1:~/SAMChainProject$ echo $start
56994717
(py2) ak2020@kubeminion1:~/SAMChainProject$ echo $end
56994817
(py2) ak2020@kubeminion1:~/SAMChainProject$ python queryReads.py chain4 chr1:${start}-${end} -dr ./myChains -ref GRCh
38_no_alt_analysis_set_GCA_000001405.15.fa
```

b)

```
A00297:35:HFKWWDSXX:2:1517:29930:2221   147     chr1    56994717        60      150M    0       56994529        -338T
CCTCTTCCGATCCAAGCCTGAATATCCAGTGGAAACTTGTAGAATAAAGCCAGGCTAAAGTGTTGTTCTCAAACATGTTTATTTGTAACAGTTTTACATGGTGTGTTACAGAAATTA
ATGGAAAATTTCTAAAAATTTTGCTGTTCTGT        <<<<<<<<<<<<<<<<<<<<<<<<<<<<<<<<<<<<<<<<<<<<<<<<<<<<<<<<<<<<<<<<<<<<<<<<<<<<<
<<<<<<<<<<<<<<<<<<<<<<<<<<<<<<<<<<<<<<<<<<<<<<<<<<<<<<<<<<<<<<<<<<<<<<<<<       MC:150M MQ:60   AS:150  XS:23   MD:15
0       NM:0    RG:HG00114_AGCTCGCT-GCAGAATC_HFKWWDSXX_L002     PG:MarkDuplicates
```

Fig S6. (a) A user intersects two bed files and query SAMchain with the resulting coordinates. (b) Output of resulting reads from this query in SAM format.

```python
if psutil.disk_usage('/').free < 2**30:
    print('ABORTING: DISK SPACE LOW')
    print('Please restart building chain for {} from read #{}'.format(data, currRead))
    return
```

Fig S7. Our codebase regularly checks for the available disk space and seals the blockchain if there is no available space. This allows users to continue adding data to their chains after increasing the available space without losing time or data.

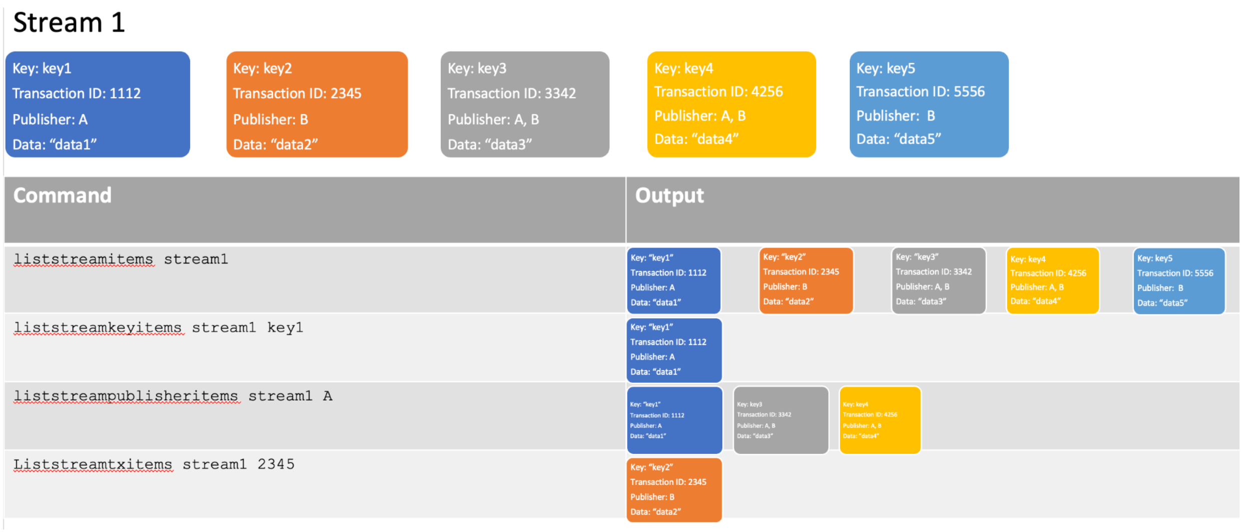

Fig S8. MultiChain specifics for "data streams". For example, we push 5 stream items to "Stream 1". Each of these items contains a key, a transaction ID, the name of the publisher, and the data itself. By using the functionalities of MultiChain, one can query the stream by listing all the items, listing items with a specific key, listing items by a specific publisher, and listing items by transaction IDs.

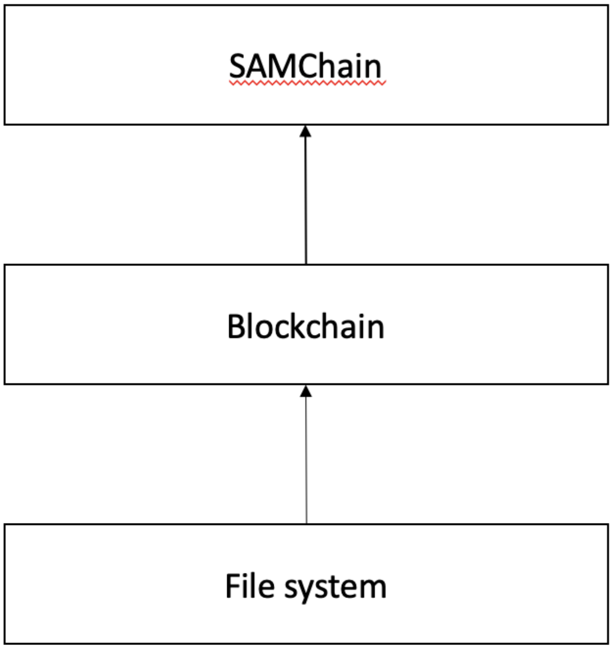

Fig S9. Blockchain is a network running on top of an existing file system, while SAMchain is an application that runs on top of a blockchain network.

Genome used: HG00114, 1000 Genomes Project
Build: GRCh38
Source of loci information: https://www.ncbi.nlm.nih.gov

Chromosome	Locus/Region	Coordinates	Length
chr1	DAB1	56994778-58546726	1.5mil bp
chr2	LRP1B	140231423-142132463	1.9mil bp
chr3	ROBO2	75906675-77649964	1.7mil bp
chr4	4p13 region	41200001-44600000	3.3mil bp
chr5	CDH18	19471296-20575886	1.1mil bp
chr6	HLA locus	28510120-33480577	4.9mil bp
chr7	CNTNAP2	146116207-148420998	2.3mil bp
chr8	CSMD1	2935353-4995035	2.0mil bp
chr9	PTPRD	8314246-10613002	2.2mil bp
chr10	PCDH15	53802771-55629182	1.8mil bp
chr11	DLG2	83455009-85628535	2.1mil bp
chr12	ANKS1B	98729904-99984773	1.2mil bp
chr13	GPC5	91398621-92867237	1.4mil bp
chr14	NRXN3	77979904-79868291	1.8mil bp
chr15	AGBL1	86079620-87031476	0.95mil bp
chr16	RBFOX1	5239752-7713343	2.4mil bp
chr17	ASIC2	33013087-34156806	1.1mil bp
chr18	DCC netrin 1 receptor	52340172-53535899	1.1mil bp
chr19	19p13.13 region	37790235-38403391	0.61mil bp
chr20	MACROD2	13995516-16053197	2.0mil bp
chr21	RUNX1	34787801-36004667	1.2mil bp
chr22	DGS/VCFS region	18924718-21111383	2.1mil bp
chrX	Dystrophin	31119219-33339460	2.2mil bp
chrY	PAR1	10001-2781479	2.7mil bp

Table S2. Details of the loci in the SAM file used for testing SAMchain.

+ advantageous for SAMchain use case
- disadvantageous for SAMchain use case

**Bitcoin**	**MultiChain**	**Ethereum**
- transactions only	+ streams- easy key:value retrieval	+ smart contracts- customizable programs
- public +private networks (better suited for public)	+ public +private networks (better suited for private)	+ public + private networks
+ on-chain data storage	+ on-chain data storage	+ on-chain data storage
- off-chain computation	- off-chain computation	+ on-chain computation
- distributed documentation	+ centralized documentation	- distributed documentation
- less commonly used as database	+ commonly used as database	- less commonly used as database

Table S3. Differences between different platforms.
