# Supplementary material for: Storing and analyzing a genome on a blockchain
Source: Genome Biol. 2022 Jun 29;23:134. doi: 10.1186/s13059-022-02699-7 (PMC9241283; doi:10.1186/s13059-022-02699-7)
Supplement: Supplementary file 2 — Additional file 2. Review History. [file 13059_2022_2699_MOESM2_ESM.docx]

**1^st^ Round**

**Reviewer 1**

The software was easy to clone and install. This software is likely to directly influence the future of work in the blockchain as applied to genomics. The question remains on whether the blockchain is likely to be a relevant or valuable technology in this field. In particular, the use of private blockchains is not necessarily in competition with public blockchains, but rather with secure and verifiable distributed databases. I am not entirely sure about the security profile that requires different use of these two data structures. The blockchain carries a great deal of infrastructural overhead, and so I think that needs to be independently justified.

The major advance in this software is query optimization and NGS compatability. In particular the ability has been added to query a genome at particular points and add SAM data. This is done through the incorporation of nested indexing. In small case use, I believe that this will be extremely valuable. However, genomic databases grow incredibly. We do not have a sense from this paper, at what scale this technology remains relevant. It is not unreasonable that a genomic center would house 100s, 1000s, 10000s, and in the future millions of genomes (especially if it's doing cancer resequencing). At what scale does this technology become unusable? Storage and time to build, seem to grow exponentially by number of reads (possibly even super-exponentially). What is the maximum number of reads (then scale that by average reads per genome) that can be stored given a petabyte of storage? I believe this is a major advance in the application of the blockchain, my concern is about the underlying technology.

One major flaw in this technology is that I don't expect it to be highly compatible with existing NGS and query pipelines. It would be best if, from the user perspective the underlying technology was transparent to the techniques being deployed. To do this, this technology should be compatible with BEDTOOLS piping. It would be far easier to deploy if a bedtools pipe could feed directly into a SAMChain query, by simply adding a SAMChain reference. This asks the question of the researcher, does the user of this software need to be a blockchain developer, or can they be a genomics user with access to a blockchain?

The methods seem appropriate. One items that I would like to see improved is a figure describing the MultiChain specifics. The paragraphs associated with the methods also need to be broken up and presented in a way that is easier to read.

The biggest problem with the results and conclusions is that it doesn't show where the range of suitability of this technology. There is a data quantity in which this technology becomes irrelevant. As such, the conclusions are incomplete. The figures describing the scale of analysis conveniently end before the chain fails. What happens if I do 10^10 reads? What happens to the system if I push it one read past where it is functional. How does this technology fail? Does it fail elegantly? If I max something out as a center, am I not able to add more to the system without buying more disks? I have real questions about the asymptotic behavior of this technology.

I believe that the details are sufficient to replicate this study and compare it with related analyses.

In general, I believe that there is broad interest in this technology and that a paper is warranted. The authors develop some innovative improvements on conventional blockchains. I have concerns about broad adoption of the technology, without a direct and in depth discussion of potential modes of failure. However, I think that this publication will be interesting to a broad audience.

**Reviewer 2**

This manuscript proposes a novel private blockchain data structure (VCFChain and SAMChain) for secure genomic analysis based on the MultiChain technology. Although blockchain is an emerging technology for secure computing, its adoption to genomic analysis is still challenging primarily due to its high overhead on data storage and queries. To address these challenges, this manuscript develops several strategies to alleviate the high demand for genomic data storage and queries on the chain. Focused on common genomic data formats (e.g. VCF, SAM), the proposed platform provides basic operations on genomic variation data and next generation sequencing data. In theory, such a platform should be extendable to secure analysis of other types of omics data. Hence, the software resulted from this manuscript will be of use to a wider community.

The manuscript is well versed, and the experiments are well designed. Experimental results support the evaluation of this platform on common operations of storing, querying, and streaming genomic data.

Although the manuscript provides extensive experimental results, it can be further improved by adding some comparative results showing some of the tradeoffs (e.g. efficient queries vs. sacrifice from the construction of SAMChain) under different scenarios (e.g. varying the number of reads with different sequence coverage or the number of genomic variants).

The figures can be enriched to include more information or improved for easy reading. For example, more annotations or descriptions can be added (Figure 1a, 1b) and some of the figures can be simplified (e.g. Figure 1c) to make them easier to follow.

**Reviewer 3**

I read the paper with great interest and it led me to dive a little into the multichain block chain solution. The well respected authors present a method that may lead to secure handling of personal genomes at time of analysis using a block chain. It is a worthwhile endeavor.

I have a list of comments below that aims to put the manuscript in a larger context to make it more interesting and refines the narrative in the interest of the reader.

Variants and aligned reads are stored compressed in a block chain ‘database’ and fetched efficiently (is the claim). Sharing comparative genomics data whilst protecting individual privacy is an extremely important topic.

When reading the abstract these thoughts came to mind:

•	Why use a block chain if hashed blocks can do?

•	Using hashes will allow for sharding - how will the block chain work for that?

•	How does this method protect individual privacy on genomic variants?

These questions are not fully answered. More below.

Major comments

1.	My main issue with the abstract is that it does not explain how privacy is protected and how a block chain actually helps. I.e., what is the real goal of the paper and how is it solved?

2.	Similar to the abstract, the introduction mostly hand waves about a potential solution. Is it worth keeping the reader in suspense? As an author I would allow lifting the veil on the actual solution earlier in the paper. My take on what the paper presents is:

The essence of the story is that the authors used a ‘novel’ approach toward using a block chain successfully to store variants and sequencer reads in a textual format. They introduced reference-genome compression and an off-the-shelve multichain approach to store large blobs, but with certain enhancements for the genomic context. I’d argue these are not really novel techniques, but they are perhaps novel in this context. The block chain prevents tampering with the data and gives granular access control. Data can be queried fairly efficiently. Even so, more work is needed to fully protect the privacy of the individual and give users autonomous control over their genomes. Also the current approach is very costly in uploading data into the block chain.

I think the paper is better served with an abstract that explains the method a little better to serve the reader. That would have helped me as a reader to appreciate the paper from the start.

3.	Then the last line of the abstract on viral sequences is puzzling. How does this relate to the rest? It looks like an opportunistic addition without further explanation. The discussion only touches on the issue of the central authority for viral strains. And yes, I also consider the GISAID situation problematic - certainly where it comes to data sharing, data mining and reproducible research. But the issue, nor the solution, is technical because the perfectly fine alternative public repositories are less used (NCBI and EBI). Your comment on viral sequence being everyone’s property is actually the point. It is proven over and over again that scientists (sadly) have no incentive to make sequences everyone’s property. It turns out this is what the sequence uploaders want: when another party accesses their GISAID data, the researchers have to sign their rights away. And that is why, for example, most downstream analysis resources (such as Nextstrain) can not share (viral) sequence data. Many of us are not happy about the situation, but a technical solution is not likely going to solve it. It is not clear to me what a block chain adds that a hashing solution (such as git, IPFS and even S3) can not solve. In the end, all you need is to track ownership. A blockchain could be a database for non-public data, but this is what we want to get away from. Right?

4.	The paper is somewhat opaque on the subject of privacy (only in the discussion encryption is mentioned). Therefore the protection of the individual through the block chain appears to be centered on the data fetching itself, or access control. Maybe I was just put on the wrong foot by ‘secure genome analysis’ in the title. The authors based their solution on MultiChain. From wikipedia:

MultiChain is an offtheshelf platform for the creation and deployment of private blockchains, either within or between organizations. It aims to overcome a key obstacle to the deployment of blockchain technology in the institutional financial sector, by providing the privacy and control required in an easytouse package.

Aha, here ‘secure’ means access control and privacy of use.

Once a blockchain is private, problems relating to scale are easily resolved, since the chain’s participants can control the maximum block size. In addition, as a closed system, the blockchain will only contain transactions which are of interest to those participants. To understand permissions in MultiChain, we begin by noting that all cryptocurrencies manage identity and security using public key cryptography.

Again, this is again about access control. So the idea is to allow a user to give certain parties access control. It is a partial solution to the larger challenge of sharing private data.

Beyond controlling access to funds, this type of cryptography enables any message to be signed by a user to prove that they own the private key corresponding to a particular address. MultiChain uses this property to restrict blockchain access to a list of permitted users, by expanding the “handshaking” process that occurs when two blockchain nodes connect:

•	Each node presents its identity as a public address on the permitted list.

•	Each node verifies that the other’s address is on its own version of the permitted list.

•	Each node sends a challenge message to the other party.

•	Each node sends back a signature of the challenge message, proving their ownership of the private key corresponding to the public address they presented.

OK, this is all about access control, which of course can be important. But there are no examples on how this access control pans out for real users.

5.	Immutable transparency of access logging is a cool feature too - only shortly mentioned in the introduction. Perhaps worth expanding on.

6.	The other part of block chains is proof of ownership. I am wondering about the real value of access control and proof of ownership. What is the chance that genomic information gets tampered with in a clinical context? Have we seen that happen?

7.	And maybe it is in the interest of an individual to change the data - for example before it gets sent to pharma or insurance companies(!?). The paper fails to discuss this aspect of mutability and how it could work.

8.	The paper claims performance (benefits). Other than a discussion and examples on the expanded Blockchain ‘streams’ I see no metrics. Oh wait, in Figure 3 there are supposed to be time metrics, but there is no caption explaining what the figure means. I have to go back to the text and it explains that these are time measurements comparing, for example, different bin sizes. There is no comparison with ‘traditional’ data fetching methods, which assumes the reader has an idea of relative performance. To be honest, I have no idea from this paper how the blockchain holds up against, say, a federated storage system, such as Arvados or even IPFS for fetching data. I suggest the final version gets proper captions and that there is some sense of relative performance against a known quantity. The text says that Fig. 3e is a time comparison but I don’t see that. I think this is not up my standards if I were the author. Please consider presenting the metrics as a comprehensive and digestable work for the reader. I am sure most readers considering this application will want to know how it holds up in practice. I am sure the authors took that into consideration for their own work (more below).

9.	In some ways the manuscript raises more questions then it answers. For example blockchains can be distributed/federated/sharded. How do we benefit from these features, how does it impact mining information on an HPC? At this point the manuscript feels incomplete. I can see blockchains becoming a part of clinical solutions, but what is necessary to get there?

10.	Furthermore, the Multichain approach here is mostly about access control. So, how does that work for the individual? Will we create an app that allows them to say who can access their data? Or is it the sequencing organisation, such as 23andme or a hospital, that gives access to the data? That is probably not what the individual wants. I.e., more questions. GA4GH is cited - these folks are creating layers and layers of APIs for access control. How can we short-cut that work using Multichains? More questions that deserve answers from the experts!

11.	Another point of note is that much of this work is based on the reference genome. Even though mapping and variant calling on the reference genome is still the dominant approach today, it is showing its short comings, and may well get replaced in the coming years. E.g. the following papers point out that new pangenome methods are emerging that are superior:

Eizenga, J. M. and Novak, A. M. and Sibbesen, J. A. and Heumos, S. and Ghaffaari, A. and Hickey, G. and Chang, X. and Seaman, J. D. and Rounthwaite, R. and Ebler, J. and Rautiainen, M. and Garg, S. and Paten, B. and Marschall, T. and Siren, J. and Garrison, E., Pangenome Graphs, Annual Review of Genomics and Human Genetics, 2020.

Sherman, R. M. and Salzberg, S. L., Pan-Genomics in the Human Genome Era, Nature Reviews Genetics, 2020.

ODGI: understanding pangenome graphs Andrea Guarracino, Simon Heumos, Sven Nahnsen, Pjotr Prins, Erik Garrison BioRXiv, 2021 doi: https://doi.org/10.1101/2021.11.10.467921

Just to elaborate a little on pangenomes:

With regard to the reference, how is it possible to capture the entire diversity of a species with an arbitrary single linear sequence? People are increasingly finding out it is not. A pangenome is a collection of genomes organized into a graph data structure to be used for holistic genetic analysis. It is hard to overstate the impact of moving from a sequence-based to a graph-based view of genomics. A traditional single sequence-based reference genome might be missing up to 10% of the genetic sequence in under-represented populations these missing sequences are the unexplored dark matter of the genetic universe. Today, all human DNA variants are identified comparing with this standard reference genome. Even though the `standard’ reference was a massive step forward at the time, it is now known to be biased and misrepresents whole populations: for example, 10% of African DNA sequence does not align to the current reference genome (see Sherman paper).

It would be interesting to bring pangenome methods to the block chain as they can be highly compressed (humans are very similar at the genomic level). Would it be possible to store and scale graphs this way? It may be worth touching on in the discussion or mentioning it as future work. the authors chose to use VCF and SAM which are highly tied to a reference genome-based approach.

13.	Likewise, the manuscript touches lightly on (homomorphic) encryption. Since you cite a paper on GWAS, you might also want to check our contribution:

Private Genomes and Public SNPs: Homomorphic Encryption of Genotypes and Phenotypes for Shared Quantitative Genetics by Mott R, Fischer C, Prins P and Davies RW, Genetics (2020) Vol 215 p. 359-372, DOI: 10.1534/genetics.120.303153 (open access)

These methods are very interesting and address the privacy protection that is missing in the blockchain approach.

14.	Another thing that puzzles me is the suggestion of introducing ssh in the discussion. Without further explanation this is very confusing. Does the blockchain not have built-in access control? Why introduce ssh as a feature? And if it is one, maybe some examples will help. Besides, I don’t know about Yale, but most clinical environments I am aware of prevent the use of ssh unless you are already on an internal network (VPN).

15.	Sorry if I appear a bit critical. The short of it is that I like the paper — it puts a stake in the ground for blockchaining large data — but I think the authors should help readers a little more and, if possible, provide more context. From the current manuscript I am not convinced this technology will take off. At best it is an exploratory use of the MultiChain implementation which won’t scale (see below).

Note that my PDF contains some notes and highlights. If you fail to see them use the free software Okular tool. And forgive me my English as I am not a native speaker.

Minor comments

16.	Some words perhaps do not belong in a science paper. I highlighted the ones that caught my eye in yellow. For example the word ‘exciting’, if used, warrants further qualification.

17.	Open data initiatives, such as OpenSNP and the Harvard public genomes, are worth mentioning because they deal with genomic variants. If data is so sensitive, what justifies their existence? Can they benefit from a more secure setup? I think they can.

18.	In the introduction the authors suggest this is the first time this approach of storing genome data in the block chain is taken. I think that is too strong a statement. I think combining IPFS hashes with blockchains is actually a better and more scalable solution. It may be worth pointing out that IPFS itself is not a block chain by some counts

https://blockchain.works-hub.com/learn/blockchain-and-interplanetary-file-system-ipfs-cec4d

but it guarantees immutable data and hashes. IPFS allows for sharding at the block level. The main problem with IPFS is that it is commonly used for public data. Even so, it is feasible to create private IPFS solutions though they are hard to share. So, this is where your block chain can shine.

Perhaps we need a hybrid solution that actually works. Storing hashed values of files in the block chain looks like the way forward to me - and it is pointed out in the text that it is exactly what other existing solutions do. I.e. the next paragraph points out ‘many up and coming’ companies store genomic data elsewhere (probably referencing from a block chain). I think this text can be a bit clearer and clearer citations would be good. And what are ‘up and coming’ companies exactly?

19.	Do all blockchains require consensus verification? I do not think so. To me a block chain is an incremental set of hashes that can not be tampered with without noticing. If I am wrong or right, please expand on that for the layman. Consensus verification is a useful extra in my mind, but not core or even critical to a block chain. Verification and node discoveries are features of particular blockchain implementations. The paper should be super clear on these definitions.

20.	Adding 10M reads (a typical NGS run for human) to the blockchain takes 55 hours. Wow. Even if that only happens once for a sequence set, I think it is a prohibitive amount of CPU time. Think about getting emergency clinical results after that lag. We should aim for less time instead of more between sequencing and results. Also CPU consumption is insensitive to current trends in climate change. There must be better methods for creating a genomic blockchain. I suggest MultiChain is not the right approach for sequence reads, though it is probably fine for efficiently stored variant calls. The discussion should bring that up. Present this paper like the pilot that it is and discuss what the remaining challenges are.

Conclusion

I think, without too much effort, the paper can expand more on these fundamental ideas and make the narrative more relevant for today’s science.

Pjotr Prins

**Authors’ response**

We sincerely thank the referees for their valuable feedback. They have significantly elevated the quality of our manuscript. In our revised manuscript, we have attempted to address all reviewers’ comments. In this document, you can find our point-by-point responses to reviewers.

Reviewer #1:

1.1 Distributed databases vs. private blockchains

Reviewer’s commentThe software was easy to clone and install. This software is likely to directly influence the future of work in the blockchain as applied to genomics. The question remains on whether the blockchain is likely to be a relevant or valuable technology in this field. In particular, the use of private blockchains is not necessarily in competition with public blockchains, but rather with secure and verifiable distribute databases. I am not entirely sure about the security profile that requires different use of these two data structures. The blockchain carries a great deal of infrastructural overhead, and so I think that needs to be independently justified.

Author’s responseWe thank the reviewer for this comment. We agree with the reviewer that there are several similarities between private blockchains and secure and verifiable distributed databases. However, there are also differences, which we tried to elaborate below and in the discussion section of the paper.

Distributed databases have been shown to be unable to prevent malicious activities when one of the participating units is hacked. Private blockchains work similar to distributed databases but they have additional levels of security. Private blockchains utilize the security processes employed by public blockchains but they do not have mathematical guarantees at the validation level. However, they still utilize the cryptographic safeguards and blockchain-specific data structures (e.g., Merkle trees) to ensure non-valid transactions are not added to the chain. Even though proof of work is not used, transactions are verified using other forms of consensus mechanisms. Thus, private blockchain provide another layer of error checking and transaction validity compared to distributed databases.

The comparison between private blockchains and distributed databases was indeed discussed in the previous blockchain literature (Kuo et al., JAMIA, 2017). In summary, private blockchains allow for decentralized database management, making them suitable for cases in which multiple parties want to collaborate without giving management control to an intermediary. They provide an immutable audit trail; provide strict data provenance and allow for better asset tracing; avoid single-point-of-failure issues, making them advantageous for preserving key records; and achieve increased security/privacy via cryptographic algorithms. However, distributed databases permit the update and deletion of records without an immutable trail (Kuo et al., JAMIA, 2017).

By contrast, the records in a blockchain (whether public or private) are protected through the block hashing mechanism, where each new block contains the information of the blocks that come before it. No such system is provided by distributed databases. Moreover and most importantly, the private blockchain described in this study can be converted into a “consortium blockchain,” in which the consensus process (i.e., proof of work in a limited way) is controlled by a pre-selected set of nodes (e.g., a set of clinician nodes), making it even more secure than distributed databases. In consortium blockchains, the read access can still be restricted to the pre-selected participants. We have added these clarifications to the introduction and discussion sections of the paper.

*Excerpt from manuscriptIn Introduction*

*Private blockchains lack mathematical guarantees at the validation level, but still utilize the cryptographic safeguards and blockchain-specific data structures (e.g., Merkle trees) to make sure non-valid transactions are not added to the chain. Although proof- of-work is not used, transactions are verified using other consensus mechanisms.*

*Private blockchain networks may be compared to distributed databases to illustrate their utility. Blockchain architecture has several advantages, as outlined by Kuo et al.11. Briefly, blockchain excels in decentralized database management, immutability (provided by the hash of a new block containing the information of the blocks that come before it), data provenance, robustness, security, and privacy. Additionally, a private blockchain may be converted into a consortium blockchain, in which consensus is controlled by a pre-selected set of nodes (i.e., proof of work in a limited way), further boosting its security.*

1.2 Scalability

Reviewer’s commentThe major advance in this software is query optimization and NGS compatability. In particular the ability has been added to query a genome at particular points and add SAM data. This is done through the incorporation of nested indexing. In small case use, I believe that this will be extremely valuable. However, genomic databases grow incredibly. We do not have a sense from this paper, at what scale this technology remains relevant. It is not unreasonable that a genomic center would house 100s, 1000s, 10000s, and in the future millions of genomes (especially if it's doing cancer resequencing). At what scale does this technology become unusable? Storage and time to build, seem to grow exponentially by number of reads (possibly even super-exponentially). What is the maximum number of reads (then scale that by average reads per genome) that can be stored given a petabyte of storage? I believe this is a major advance in the application of the blockchain, my concern is about the underlying technology.

Author’s responseWe thank the reviewer for this question.

We think that scalability will not be a problem moving forward because 1- the genome is fixed in size and we showed that it can be stored on the blockchain with the current data storage options we have; 2- the cost of pushing data, although seems high, is a one-time cost and is attainable, as we showed with our new parallel system; 3- it is designed to hold a single human genome; 4- for storing many genomes, we would create many SAMchains operated by the owner of the genomes.

Please note that, in our study, each private blockchain network corresponds to a single genome owned by the individual to which the genome belongs. In addition, a single genome will always have a fixed size (for example, 30x WGS will always have 400-600 million reads based on the read length). Therefore, we are confident that neither the storage nor the time required to push the data (one time cost) will be an issue moving forward since every blockchain network is designed to contain a single genome. If we were to store 100 million genomes, then we would create 100 million private blockchain networks. That is, each network only has to scale to the size of a single genome, which is a bounded entity. The sequencer node does not have to store these genomes; their job is to push the data directly to the private network, after which they can unsubscribe from the network. For more genomes, there will be more private blockchain networks to which researchers and clinicians can subscribe. They will only store the data if they subscribe to that particular network. This allows distribution of the storage load across the bigger network with a large number of patients. This is possible because, in blockchain, one can run multiple blockchains in parallel, between the same servers, and even share identities across them by using the same addresses. The only storage problem would arise if a “researcher” or a “clinician” node would like to connect to hundreds of small private blockchain networks and perform simultaneous queries. In that case, they would have to sync all the information in all of these networks.

However, in Figure 2b, we have shown that MultiChain syncs the data in the nodes in such a way that it is compressed and indexed; therefore, the storage increase will not scale 1:1 with the size of the genomes. We demonstrated that each node syncs about 35% of the data that reside in the owner node, which is 0.00044 petabyte per genome. This means that a “researcher” or “clinician” node can subscribe to ~2,273 genomes (i.e., private blockchains) simultaneously given 1 petabyte of storage. Moreover, our system is highly parallelizable, i.e., a researcher can subscribe to the blockchain using multiple nodes and can query multiple genomes in parallel.

We have also now performed a more detailed scalability analysis where we store data from 100 millions of reads. Time to build the chain appears to grow linearly with the number of reads, shown in Figure 2C (note that the figure is in log scale). The 100 millions of reads take 211 GB of space. Considering a 30x whole-genome sequencing (WGS) dataset is about 400-600 million reads depending on the read length, it would take around 0.001266 petabyte of storage. Note that each genome sequencing takes a limited amount of space because the size of these reads are well defined per 30X sequencing.

We have now made this clear in the manuscript.

We have also improved our codebase such that we can build the chain in parallel by taking advantage of multiple CPU cores. This improved the chain building time by up to 5 times.

*Excerpt from manuscriptIn “Discussion”:*

*Please note that, in our study, each private blockchain network corresponds to a single genome owned by the individual to which the genome belongs. In addition, a single genome will always have an approximately fixed size (for example, 30X whole-genome sequencing will always have 400-600 million reads based on the read length). That is, genomes from millions of users translate into millions of private blockchain networks such that each network must scale only to a fixed, single genome size. Therefore, we believe that neither storage nor the time to push the data (one time cost) will be an issue moving forward if this technology is widely adopted. Moreover, researchers can subscribe to multiple networks at once to query data in parallel from multiple genomes.*

*To summarize briefly, we think that the scalability will not be a problem moving forward, because 1- the genome is fixed in size and we showed that it can be stored on the blockchain with the current data storage options we have; 2- the cost of pushing data, although seems high, is a one-time cost and is attainable, as we showed with our new parallel system; 3- it is designed to hold a single human genome; 4- for storing many genomes, we would create many SAMchains operated by the owner of the genomes. In “SAMchain provides a platform for storing next-generation sequencing (NGS) data in blockchain”*

*Since each read in a SAM file is independent and the streams to store the reads are automatically determined by our algorithm based on its coordinates, we can use multiple nodes to push the data in a SAM file in a parallel fashion. To this end, we parallelized our codebase and showed that we can improve data insertion time by 5- fold using 10 CPUs at the same time (Figure 2c). We also found that the 5-fold increase is the limit of the possible speedup because of the transaction latency in the blockchain. (This latency is due to safeguards such as proof of authority.) As an illustration, we tried to push 100,000,000 reads into the SAMchain, which took ~100 hours using 10 CPUs. Note that there is a balance between how much data is stored and how quickly the data can be queried. As the data storage is needed only once, our optimizations are geared towards efficient queries. This is different than current blockchain genomic data storage options, where the data storage is optimized and outsourced to other distributed systems such as IPFS, hence there is no option for querying and analyzing the data on the chain.*

*Figure 2. SAMchain performance. (a) Before inserting SAM data to a SAMchain, we remove the sequence and quality strings. In plain text, we measured the storage gain by removing these fields. (b) The total network storage required for a SAMchain storing ~10 million reads. (c) Total time to insert data to SAMchain as a function of number of reads when different number of CPUs are used. (d) Storage (compared to a SAM file), time (for a single CPU), and memory used to build a SAMchain.*

1.3 Compatibility

Reviewer’s commentOne major flaw in this technology is that I don't expect it to be highly compatible with existing NGS and query pipelines. It would be best if, from the user perspective the underlying technology was transparent to the techniques being deployed. To do this, this technology should be compatible with BEDTOOLS piping. It would be far easier to deploy if a bedtools pipe could feed directly into a SAMChain query, by simply adding a SAMChain reference. This asks the question of the researcher, does the user of this software need to be a blockchain developer, or can they be a genomics user with access to a blockchain?

Author’s responseWe thank the reviewer for this insightful question.

It is indeed quite easy to build BEDTOOLs piping to feed into SAMChain. We developed SAMchain using Python. We added several wrappers around MultiChain API such that users do not have to know the underlying blockchain technology. SAMchain and SCtools functionalities are used by invoking Python scripts in the command line. In addition to BEDTOOLS, any other software (e.g., BCFtools) or programming language (e.g., AWK) can be piped into SAMChain.

We now include some examples of how piping works in the supplementary information. For example, if a user would like to create a bed file from the bam file built by SCtools, they can simply use python buildBAM.py <my-chain-name> -dr <datadir> -bam <filename>.bam -ref <ref- genome-filename> | bedtools bamtobed -i stdin

Supplementary Information contains a more elaborate example (see below). Researchers or users of this software do not need to be experts in blockchain. The MultiChain installation only takes a simple UNIX command. The rest of the operations regarding the generation of chain, inserting data, and querying are all written in Python and only require a single line of command, as is shown in the example below.

*Excerpt from manuscriptIn Results:*

*SAMchain is user friendly and easy to use by users with no blockchain knowledge The design of SAMChain and SCtools provides several user-friendly options to scientists with no blockchain background. The modules in SAMchain and SCtools are developed in Python and allows for storing and querying of data using a command line. This allows users to combine the functionalities of our tools with commonly used command line bioinformatics tools such as BEDtools (Supplementary Figure 6). Our codebase also alerts users about potential data storage issues: It regularly checks the remaining storage space during the data storage process. If there is not enough space to add the next read to the chain, it seals the chain and returns a storage error with an indication where the chain has failed. With this, users do not have to rebuild the chain but can continue adding to the chain from where it failed after increasing the available storage (Supplementary Figure 7). Furthermore, our system works well in a high performance computing (HPC) setting. This means, as long as access is granted, scientists can easily install the MultiChain client and SAMChain app and connect to an existing private blockchain network to mine genomics data by using their institution's existing HPC infrastructure.*

*In SI:*

*Compatibility with other NGS Software*

*We developed SAMchain using Python. We added several wrappers around MultiChain API such that users do not have to know the underlying blockchain technology. SAMchain and SCtools functionalities are used by invoking Python scripts in the command line. In addition to BEDTOOLS, any other software (e.g., BCFtools) or programming language (e.g., AWK) can be piped into SAMChain. SI Figure 6 is an example of how SAMchain might be used with BEDtools.*

*1.4 Figure for MultiChain specifics*

*Reviewer’s commentThe methods seem appropriate. One items that I would like to see improved is a figure describing the MultiChain specifics. The paragraphs associated with the methods also need to be broken up and presented in a way that is easier to read.*

*Author’s responseWe thank the reviewer for this suggestion.*

*We have now added a figure to the supplement explaining the MultiChain specifics. We have also broken up the methods section for better readability.*

*Excerpt from manuscriptIn Supplementary Info:*

1.5 Scalability and failure of the system

Reviewer’s commentThe biggest problem with the results and conclusions is that it doesn't show where the range of suitability of this technology. There is a data quantity in which this technology becomes irrelevant. As such, the conclusions are incomplete.

The figures describing the scale of analysis conveniently end before the chain fails. What happens if I do 10^10 reads? What happens to the system if I push it one read past where it is functional. How does this technology fail? Does it fail elegantly? If I max something out as a center, am I not able to add more to the system without buying more disks? I have real questions about the asymptotic behavior of this technology.

Author’s responseWe thank the reviewer for this question. We have partially responded to this question in 1.2 in relation to the scalability of SAMchain. In particular in Figure 2c, we have now performed a more detailed scalability analysis where we store data from 100 millions of reads, which is about 1/6th of a genome with 30x coverage.

We have also improved our codebase such that we can now build the chain in parallel since each chromosome is indexed in different data streams. This improved the chain building time by 5 times.

Finally, we have added a functionality to the codebase: It now regularly checks the remaining storage space. If there is not enough space to add the next read to the chain, it seals the chain and returns a storage error with an indication where the chain has failed. With this, users do not have to rebuild the chain but can continue adding to the

*Excerpt from manuscriptSee Excerpt in 1.2*

*In SAMchain is user friendly and easy to use by users with no blockchain knowledge*

*Our codebase also alerts users about potential data storage issues: It regularly checks the remaining storage space during the data storage process. If there is not enough space to add the next read to the chain, it seals the chain and returns a storage error with an indication where the chain has failed. With this, users do not have to rebuild the chain but can continue adding to the chain from where it failed after increasing the available storage (Supplementary Figure 7).*

1.6 Sufficiency of details

Reviewer’s commentI believe that the details are sufficient to replicate this study and compare it with related analyses.

Author’s responseWe thank the reviewer for this comment.

1.7 Broad interest

Reviewer’s commentIn general, I believe that there is broad interest in this technology and that a paper is warranted. The authors develop some innovative improvements on conventional blockchains. I have concerns about broad adoption of the technology, without a direct and in depth discussion of potential modes of failure. However, I think that this publication will be interesting to a broad audience.

Author’s responseWe thank the reviewer for this comment. We hope that our new discussion, analysis on scalability, and additional functions are satisfactory.

Reviewer #2:

2.1 Overall comments

Reviewer’s commentThis manuscript proposes a novel private blockchain data structure (VCFChain and SAMChain) for secure genomic analysis based on the MultiChain technology. Although blockchain is an emerging technology for secure computing, its adoption to genomic analysis is still challenging primarily due to its high overhead on data storage and queries. To address these challenges, this manuscript develops several strategies to alleviate the high demand for genomic data storage and queries on the chain. Focused on common genomic data formats (e.g. VCF, SAM), the proposed platform provides basic operations on genomic variation data and next generation sequencing data. In theory, such a platform should be extendable to secure analysis of other types of omics data. Hence, the software resulted from this manuscript will be of use to a wider community.

Author’s responseWe thank the reviewer for this comment.

2.2 Writing

Reviewer’s commentThe manuscript is well versed, and the experiments are well designed. Experimental results support the evaluation of this platform on common operations of storing, querying, and streaming genomic data.

Author’s responseWe thank the reviewer for this comment.

2.3 Trade-off calculations

Reviewer’s commentAlthough the manuscript provides extensive experimental results, it can be further improved by adding some comparative results showing some of the tradeoffs (e.g. efficient queries vs. sacrifice from the construction of SAMChain) under different scenarios (e.g. varying the number of reads with different sequence coverage or the number of genomic variants).

Author’s responseWe thank the reviewer for this comment.

Our manuscript currently includes this type of trade-off analysis. For example, please see Figure 3. Figure 3c shows that querying becomes less efficient as the chain storage capacity increases and that construction time also increases with storage.

Figure 3f illustrates that the time to build a BAM file increases with chain storage.

Figures 3b and 3d show the performance when increasing the number of reads stored in a stream. Streams are binned based on genomic location, so this is analogous to varying the sequence coverage (in other words, a greater sequence coverage will lead to greater numbers of reads in each stream, the effect of which is shown in our figures).

One should certainly be aware of these tradeoffs when considering the design of SAMChain. However, we also show in Figure 3d the benefit of our approach - binning streams by genomic position - as compared to a single stream approach. For example, we showed that if we increase the bin sizes, the amount of gain we will have in the storage is around ~6% of the original storage need (55.5 GB vs. 52.5 GB). But, the amount of gain we make in query time is ~40-fold faster (10 seconds vs. 400 seconds).

*Excerpt from manuscriptIn “SCtools can query NGS properties directly from SAMchain” In Figure 3c, we investigated the impact of changing the SAMchain bin size for a fixed number of reads stored (~10 million). We found that increasing the bin size increases the query time, which we expected because a larger bin size also contains a higher number of reads relative to a smaller bin size. However, increasing the bin size also decreases the total number of streams, which reduces the time required to build the SAMchain (Figure 3c). In Figure 3d, we measured query time for a SAMchain storing a fixed number of reads (~10 million) as a function of the range of genomic coordinates queried. We found that query time increases linearly with an increasing range queried. This shows that if we increase the bin sizes, we can reduce storage size by ~6% of the original storage need (55.5 GB vs. 52.5 GB). On the other hand, the time to query data becomes ~40-fold slower (10 seconds vs. 400 seconds). These trade-offs can help dictate whether a particular use case is appropriate for SAMchain.*

2.4 Figures

Reviewer’s commentThe figures can be enriched to include more information or improved for easy reading. For example, more annotations or descriptions can be added (Figure 1a, 1b) and some of the figures can be simplified (e.g. Figure 1c) to make them easier to follow.

Author’s responseWe thank the reviewer for this suggestion. We have now added more details to Figure 1a so it is self-contained. We also annotated Figure 1b with more details. We simplified the steps in Figure 1c so it is clearly understandable.

*Excerpt from manuscript*

*Figure 1. SAMchain design and implementation. (a) Overview of the SAMchain network ecosystem. The network consists of owner, sequencer, clinician, and researcher nodes. The owner node builds the SAMchain and the sequencer node accesses the chain and inserts SAM data into it. The clinician and researcher nodes access the SAMchain and analyze the on-chain SAM data. (b) Details of data storage in SAMchain. A read is typically stored in a SAM file containing several features. Our data structure is organized by genomic location. A single stream, named metaData, contains all of the header data and other chain info. Many other streams serve as bins by genomic location, and hold the SAM feature data and MODCIGAR. A FLANK feature is used to indicate whether a read’s position spans two consecutive bins. Stream items correspond to a single read. A single stream, named unmappedANDcontigs stores unmapped reads and contigs. (c) Overview of the query process. Upon querying a genomic location, our algorithm searches through the binned streams to obtain the SAM data and MODCIGAR features corresponding to the specified location. These data, in combination with a reference genome, yield a complete SAM read. Our algorithms and stream-based data structures are built on top of MultiChain, which provides the underlying blockchain, stream design, and network configuration.*

Reviewer #3:

3.1 Overall Comments

Reviewer’s commentBlockchain for genomics

I read the paper with great interest and it led me to dive a little into the multichain block chain solution. The well respected authors present a method that may lead to secure handling of personal genomes at time of analysis using a block chain. It is a worthwhile endeavor.

I have a list of comments below that aims to put the manuscript in a larger context to make it more interesting and refines the narrative in the interest of the reader. Variants and aligned reads are stored compressed in a block chain ‘database’ and fetched efficiently (is the claim). Sharing comparative genomics data whilst protecting individual privacy is an extremely important topic.

When reading the abstract these thoughts came to mind:

• Why use a block chain if hashed blocks can do?

• Using hashes will allow for sharding - how will the block chain work for that?

• How does this method protect individual privacy on genomic variants?

These questions are not fully answered. More below.

Author’s responseWe thank the reviewer for the valuable comments and for taking the time to delve into the details of the blockchain architecture. Here we tried to answer the reviewer's questions in detail. We were not able to add all of the suggested nuances to the abstract due to the 100 word limit. However, we made an effort to address them all in the introduction and discussion. In summary, we have changed the title, abstract, introduction, and discussion.

To answer the particular questions here:

●We used blockchain instead of hashed blocks because the data stream property of MultiChain allows us to do specific queries within a block by being able to hash every single transaction (i.e., line of data) in a principled way. With hashed blocks, we can retrieve the data in a block altogether but cannot index and search, for example, a specific read in NGS data. Most importantly, blockchain provides immutability. This is provided by blockchain hashing mechanisms that other systems do not have. In blockchain, the hash of every block contains the information stored in all of the blocks that come before it. This makes it extremely difficult to change any information in the previous blocks, making it more secure to tampering.

●It is not possible to shard an individual blockchain, because the hash of the blockchain depends on all the context in the blocks, which makes the content secure and hence different from a regular database. However, since a single blockchain corresponds to a single genome, one can certainly run multiple blockchains in parallel, between the same servers, and even share identities across them by using the same addresses.

●The privacy is protected by giving the control of the data to the individuals whose biosamples are sequenced, which allows them to control who and when the data can be accessed as opposed to giving a company or an institution control of the access. The goal of the paper is not to provide privacy to data that was held by a third party, but to provide security and data ownership of the data to the individuals. We want to make it clear that security is different from privacy. Privacy is one’s right to control one’s own information, while security refers to how the information is protected. We provide secure storage and give full control of data to the individuals in this system. They then have control over their own privacy.

*Excerpt from manuscriptAbstract:*

*There are major efforts underway to make genome sequencing a routine part of clinical practice. A critical barrier to these is achieving practical solutions for data ownership and integrity. Blockchain provides solutions to these challenges in other realms (e.g. finance). However, its use in genomics is stymied due to the difficulty in storing large- scale data on chain, slow transaction speeds, and limitations on querying. To overcome these roadblocks, we developed a private blockchain network to store genomic variants and reference-aligned reads on-chain. It uses nested database indexing with an accompanying tool suite to rapidly access and analyze the data.*

Major comments

3.2 Abstract

Reviewer’s comment My main issue with the abstract is that it does not explain how privacy is protected and how a block chain actually helps. I.e., what is the real goal of the paper and how is it solved?

Author’s responseThe goal of the paper is not to provide privacy to data that is held by a third party, but to provide security and data ownership of the data to the individuals using blockchain technology. We have now made this clear in the abstract. Please also note that there is a 100 word limit in the abstract mandated by the Journal.

*Excerpt from manuscriptAbstract:*

*There are major efforts underway to make genome sequencing a routine part of clinical practice. A critical barrier to these is achieving practical solutions for data ownership and integrity. Blockchain provides solutions to these challenges in other realms (e.g. finance). However, its use in genomics is stymied due to the difficulty in storing large- scale data on chain, slow transaction speeds, and limitations on querying. To overcome these roadblocks, we developed a private blockchain network to store genomic variants and reference-aligned reads on-chain. It uses nested database indexing with an accompanying tool suite to rapidly access and analyze the data.*

3.3 Introduction

Reviewer’s commentSimilar to the abstract, the introduction mostly hand waves about a potential solution. Is it worth keeping the reader in suspense? As an author I would allow lifting the veil on the actual solution earlier in the paper. My take on what the paper presents is:

The essence of the story is that the authors used a ‘novel’ approach toward using a block chain successfully to store variants and sequencer reads in a textual format. They introduced reference-genome compression and an off-the-shelve multichain approach to store large blobs, but with certain enhancements for the genomic context. I’d argue these are not really novel techniques, but they are perhaps novel in this context. The block chain prevents tampering with the data and gives granular access control. Data can be queried fairly efficiently. Even so, more work is needed to fully protect the privacy of the individual and give users autonomous control over their genomes. Also the current approach is very costly in uploading data into the block chain.

I think the paper is better served with an abstract that explains the method a little better to serve the reader. That would have helped me as a reader to appreciate the paper from the start.

Author’s responseWe thank the reviewer for this comment. We have now rewritten the abstract and changed the introduction to reflect the reviewer’s suggestions.

*Excerpt from manuscriptAbstract:*

*There are major efforts underway to make genome sequencing a routine part of clinical practice. A critical barrier to these is achieving practical solutions for data ownership and integrity. Blockchain provides solutions to these challenges in other realms (e.g. finance). However, its use in genomics is stymied due to the difficulty in storing large- scale data on chain, slow transaction speeds, and limitations on querying. To overcome these roadblocks, we developed a private blockchain network to store genomic variants and reference-aligned reads on-chain. It uses nested database indexing with an accompanying tool suite to rapidly access and analyze the data.*

*In Introduction:*

*Ideally, individuals would retain ownership and be able to benefit from their own data. This enables them to control the right to share (privacy) and how to share (security) their own genetic data. Yet, the storage and analysis infrastructure to achieve this goal is lacking.*

*....*

*Note, many up and coming biotech companies offer blockchain-based solutions for genomic data sharing (see Supplementary Information for more about these companies and blockchain technology). Because of the technical challenges related to storing genomes in a blockchain infrastructure, many blockchain-genomics companies store the genomic data elsewhere, such as in Blockstack or the InterPlanetary File System (IPFS) 7–9 and use blockchains as a log-keeping infrastructure (see Supplementary Information for more about the difference). In particular, IPFS is a decentralized file system that uses a distributed hash table for easy access to the shared files in an effort to create a resilient system for file storage and sharing.*

*....*

*An ideal implementation of personal genomic data storage would 1) protect from loss and manipulation, 2) provide appropriate access to clinicians and biomedical researchers, and 3) allow individuals control over their own genomic data. Private blockchains lack mathematical guarantees at the validation level, but still utilize the cryptographic safeguards and blockchain-specific data structures (e.g., Merkle trees) to make sure non-valid transactions are not added to the chain. Although proof-of-work is not used, transactions are verified using other consensus mechanisms. Private blockchain networks may be compared to distributed databases to illustrate their utility. Blockchain architecture has several advantages, as outlined by Kuo et al.11. Briefly, blockchain excels in decentralized database management, immutability (provided by the hash of a new block containing the information of the blocks that come before it), data provenance, robustness, security, and privacy. Additionally, a private blockchain may be converted into a consortium blockchain, in which consensus is controlled by a pre-selected set of nodes (i.e., proof of work in a limited way), further boosting its security.*

*...*

*In this study, we present the first open-source, proof-of-concept private blockchain network, which allows efficient storage and retrieval of personal genetic variants [often stored as variant call format (VCF) files] and raw genomic reads [often stored as sequence alignment map (SAM) files] 12,13.*

3.4 Viral sequencing

Reviewer’s commentThen the last line of the abstract on viral sequences is puzzling. How does this relate to the rest? It looks like an opportunistic addition without further explanation. The discussion only touches on the issue of the central authority for viral strains. And yes, I also consider the GISAID situation problematic - certainly where it comes to data sharing, data mining and reproducible research. But the issue, nor the solution, is technical because the perfectly fine alternative public repositories are less used (NCBI and EBI). Your comment on viral sequence being everyone’s property is actually the point. It is proven over and over again that scientists (sadly) have no incentive to make sequences everyone’s property. It turns out this is what the sequence uploaders want: when another party accesses their GISAID data, the researchers have to sign their rights away. And that is why, for example, most downstream analysis resources (such as Nextstrain) can not share (viral) sequence data. Many of us are not happy about the situation, but a technical solution is not likely going to solve it. It is not clear to me what a block chain adds that a hashing solution (such as git, IPFS and even S3) can not solve. In the end, all you need is to track ownership. A blockchain could be a database for non-public data, but this is what we want to get away from. Right?

Author’s responseWe agree with the reviewer on this aspect and we have now removed the sections related to viral sequencing. We think this makes the paper less confusing and more accurate.

3.5 privacy vs. security

Reviewer’s comment The paper is somewhat opaque on the subject of privacy (only in the discussion encryption is mentioned). Therefore the protection of the individual through the block chain appears to be centered on the data fetching itself, or access control. Maybe I was just put on the wrong foot by ‘secure genome analysis’ in the title.

The authors based their solution on MultiChain. From wikipedia:

MultiChain is an offtheshelf platform for the creation and deployment of private blockchains, either within or between organizations. It aims to overcome a key obstacle to the deployment of blockchain technology in the institutional financial sector, by providing the privacy and control required in an easytouse package.

Aha, here ‘secure’ means access control and privacy of use.

Once a blockchain is private, problems relating to scale are easily resolved, since the chain’s participants can control the maximum block size. In addition, as a closed system, the blockchain will only contain transactions which are of interest to those participants. To understand permissions in MultiChain, we begin by noting that all cryptocurrencies manage identity and security using public key cryptography. Again, this is again about access control. So the idea is to allow a user to give certain parties access control. It is a partial solution to the larger challenge of sharing private data.

Beyond controlling access to funds, this type of cryptography enables any message to be signed by a user to prove that they own the private key corresponding to a particular address. MultiChain uses this property to restrict blockchain access to a list of permitted users, by expanding the “handshaking” process that occurs when two blockchain nodes connect:

• Each node presents its identity as a public address on the permitted list.

• Each node verifies that the other’s address is on its own version of the permitted list.

• Each node sends a challenge message to the other party.

• Each node sends back a signature of the challenge message, proving their ownership of the private key corresponding to the public address they presented.

OK, this is all about access control, which of course can be important. But there are no examples on how this access control pans out for real users.

Author’s responseWe thank the reviewer for this comment.

We first would like to clarify that secure and private mean different things. For example, security in blockchain is inherent in its block hashing property (i.e., the new block’s hash contains the information of all the blocks before it). This makes the system secure in terms of its immutability. We tried to make the distinction between security and privacy clear. We have also removed the word secure from the title. Privacy in the SAMChain design comes into play with access control. However, it is different from the access control systems embedded in, for example, dbGAP or EGA, where a third party controls the access to a genome, but the patient from which the data are taken has no say on this access control. In SAMChain, a patient or a curious user of a genetic genealogy owns the data and allows the others access. The control of access and who queries what is hashed via cryptographic functions provided by blockchain, which makes it more secure than other alternative privately-owned distributed databases. Access control is inherent in MultiChain, in which the owner of the network can grant access to a user and make the user part of the blockchain. This is done by assigning a wallet address to the user. Then all the transactions the user does are carefully documented in the blockchain. We have now made this clear in the manuscript and changed the title accordingly.

*Excerpt from manuscriptNew title:*

*Storing and analyzing a genome on a blockchain*

*In Introduction:*

*....Ideally, individuals would retain ownership and be able to benefit from their own data. This enables them to control the right to share (privacy) and how to share (security) their own genetic data.....*

*In “Private blockchain networks can help with control of data “*

*... MultiChain, the technology that SAMChain is built upon, provides access control, in which the owner of the blockchain network can grant permission to a user and make the user part of the blockchain network. It also provides the option of granting only partial access to the chain...*

*In Discussion*

*We envision a real-world scenario in which individuals create private blockchains to store their personal genomes to share with their healthcare providers and biomedical researchers. Simply with intrinsic access control and security properties, healthcare providers and associated genetics researchers can stream or query patients’ genomes. For example, every time a piece of data is encrypted in a shared server or cloud, there is a potential for it to be corrupted, especially if the data are extremely large such as genomic data. Moreover, traditional access-control mechanisms such as dbGAP or EGA leave the research participant or patient outside of the decision-making process. Our private blockchain network reduces not only the risk of data corruption, but also non-permissioned access to private data and gives the control to the actual owner of the data. Blockchain provides immutability such that the data cannot be altered, whether intentionally or accidentally. This is an important aspect of the system not only for personal, private data but also for large-scale open genomic data. Large-scale open data such as those from the 1,000 Genomes Project or the Personal Genomes Project, especially in centralized data storage systems, are vulnerable to corruption and tampering. Moreover, our blockchain-based system can also be combined with blockchain-based data access auditing solutions 5,25 to provide further control and auditing of access in a more efficient way.*

3.6 access logging

Reviewer’s comment Immutable transparency of access logging is a cool feature too - only shortly mentioned in the introduction. Perhaps worth expanding on.

Author’s responseWe thank the reviewer for this comment. Every time access is granted and made, it is logged in the system in an immutable way with the accessing node’s wallet ID. We have now mentioned this in the paper as well. Moreover, we are interested in intrinsic access logging. We previously developed a system to keep and query the access logs in an immutable way (Gursoy et al., BMC Medical Genomics, 2020). In the future, we are interested in combining the current study with our BMC Medical Genomics implementation by creating a separate system to keep the access logs in a stream, because it is easier to query streams than the intrinsic access logs. We have now mentioned this in the discussion.

*Excerpt from manuscriptIn Introduction:*

*Moreover, every time a user subscribes to the network and pushes data to the network, it is recorded in the blockchain with precise timestamps.*

*In Discussion:*

*Moreover, our blockchain-based system can also be combined with blockchain-based data access auditing solutions (cite idash papers) to provide further control and auditing of access in a more efficient way.*

3.7 proof of ownership

Reviewer’s commentThe other part of block chains is proof of ownership. I am wondering about the real value of access control and proof of ownership. What is the chance that genomic information gets tampered with in a clinical context? Have we seen that happen?

Author’s responseIn a public blockchain, there is no need for proof of ownership because the entire blockchain is owned by the owner of the data, therefore there is a strong access control mechanism. Access control to the genomic data is immensely important. It is about not only preventing the data from being tampered with but also knowing who accesses the data for what purposes.

Another issue in non-blockchain-based systems is data corruption. For example, every time the data are encrypted and decrypted in a shared server or cloud, there is high potential for the data to be corrupted, especially for very large datasets such as genomes from millions of people. There are, of course, various integrity checks such as MD5cheksum; but there is no mechanism to correct for the already corrupted data. Blockchain also allows us to protect the integrity of the data from corruption, so what blockchain provides is not only about data tampering. For example, for datasets such as those from the 1000 Genomes or Personal Genome Project (PGP), data are used for years and data integrity becomes an important issue. As the size of the data increases, making sure there is no corruption of the data becomes more difficult. Note that this issue has nothing to do with privacy since these are totally open data sets. We have now made this property more clear in the Discussion.

In terms of genomic information being tampered with, there was an attack in 2019. Researchers from Sandia Labs attacked the genome alignment tool BWA to tamper with the genetic variants that were obtained from patient genomes. This attack received extensive attention from the media:

http://www2.computerworld.com.au/article/663645/vulnerability-allowed-attackers-edit-

patient-gene-sequence-records/,

<https://newsreleases.sandia.gov/genomic_cybersecurity/>

*In Discussion:*

*Blockchain provides immutability such that the data cannot be altered, whether intentionally or accidentally. This is an important aspect of the system not only for personal, private data but also for large-scale open genomic data. Large-scale open data such as those from the 1,000 Genomes Project or the Personal Genomes Project, especially in centralized data storage systems, are vulnerable to corruption and tampering. Moreover, our blockchain-based system can also be combined with blockchain-based data access auditing solutions 5,25 to provide further control and auditing of access in a more efficient way.*

3.8 mutability

Reviewer’s comment7. And maybe it is in the interest of an individual to change the data - for example before it gets sent to pharma or insurance companies(!?). The paper fails to discuss this aspect of mutability and how it could work.

Author’s responseOnce the individual has control over the data, they have all the power to append changed data to the blockchain and give access to pharmaceutical or insurance companies only to the changed or noised part of the data. One can create any type of new data streams to selectively add tampered data to disseminate with industry while keeping other streams with the immutable data for the clinicians. For example, in MultiChain, permissions can be made temporary or limited to a specific range of block numbers so that they are available only to transactions in those blocks.

We have now added a sentence on this to the discussion.

*Excerpt from manuscriptIn Private blockchain networks can help with control of data MultiChain, the technology that SAMChain is built upon, provides access control, in which the owner of the blockchain network can grant permission to a user and make the user part of the blockchain network. It also provides the option of granting only partial access to the chain.*

3.8 IPFS vs. SAMchain

Reviewer’s commentThe paper claims performance (benefits). Other than a discussion and examples on the expanded Blockchain ‘streams’ I see no metrics. Oh wait, in Figure 3 there are supposed to be time metrics, but there is no caption explaining what the figure means. I have to go back to the text and it explains that these are time measurements comparing, for example, different bin sizes. There is no comparison with ‘traditional’ data fetching methods, which assumes the reader has an idea of relative performance. To be honest, I have no idea from this paper how the blockchain holds up against, say, a federated storage system, such as Arvados or even IPFS for fetching data. I suggest the final version gets proper captions and that there is some sense of relative performance against a known quantity. The text says that Fig. 3e is a time comparison but I don’t see that. I think this is not up my standards if I were the author. Please consider presenting the metrics as a comprehensive and digestable work for the reader. I am sure most readers considering this application will want to know how it holds up in practice. I am sure the authors took that into consideration for their own work (more below).

Author’s response We thank the reviewer for this comment.

First, we want to note that we provided captions of the figures; they were at the end of the paper per journal’s format and perhaps were not easily accessible. We agree that there needs to be more intuitive comparisons in the paper. To this end, we have now added a paragraph explaining the query time complexity and direct comparison to the plaintext counterpart tool SAMtools.

We think that there might be a misunderstanding on how our system works. SAMChain indexes the data read by read and it uses nested indexing schemes even within a read. That is, one can query and stream the data with many properties in the read. For example, one can ask a question about whether a certain mutation in a single location of the genome exists in the stored genome. One can also return the depth of sequencing in a particular location, or ask for the reads by their readname. None of these features are provided by IPFS or Arvados so we cannot make a comparison for these queries. IPFS and Arvados are infrastructures while SAMchain is an application, so one can implement SAMchain on top of the existing IPFS and Arvados systems.

IPFS and Arvados do not have hashing for information in the file, i.e., it is not possible to perform the granular queries that can be done using SAMchain. They are used for large-scale data sharing but not micro- scale querying. In addition, Arvados is designed for bioinformatics researchers and has a complicated codebase. It is not clear how a patient can easily navigate and perform access control for scientists and cliniciansa. In summary, IPFS allows encrypted data storage in a distributed way, and Arvados is a platform that allows for the retrieval and computation of the data by its hash; however granular key-data querying is not provided. Most importantly, the immutability that comes with blockchain due to each block’s hash containing the information that comes before it is not possible on Arvados with the IPFS.

We have now added a small section describing these differences.

*Excerpt from manuscript*

*Figure Captions:*

*Figure 1. SAMchain design and implementation. (a) Overview of the SAMchain network ecosystem. The network consists of owner, sequencer, clinician, and researcher nodes. The owner node builds the SAMchain and the sequencer node accesses the chain and inserts SAM data into it. The clinician and researcher nodes access the SAMchain and analyze the on-chain SAM data. (b) Details of data storage in SAMchain. A read is typically stored in a SAM file containing several features. Our data structure is organized by genomic location. A single stream, named metaData, contains all of the header data and other chain info. Many other streams serve as bins by genomic location, and hold the SAM feature data and MODCIGAR. A FLANK feature is used to indicate whether a read’s position spans two consecutive bins. Stream items correspond to a single read. A single stream, named unmappedANDcontigs stores unmapped reads and contigs. (c) Overview of the query process. Upon querying a genomic location, our algorithm searches through the binned streams to obtain the SAM data and MODCIGAR features corresponding to the specified location. These data, in combination with a reference genome, yield a complete SAM read. Our algorithms and stream-based data structures are built on top of MultiChain, which provides the underlying blockchain, stream design, and network configuration.*

*Figure 2. SAMchain performance. (a) Before inserting SAM data to a SAMchain, we remove the sequence and quality strings. In plain text, we measured the storage gain by removing these fields. (b) The total network storage required for a SAMchain storing ~10 million reads. (c) Total time to insert data to SAMchain as a function of number of reads when different number of CPUs are used. (d) Storage (compared to a SAM file), time (for a single CPU), and memory used to build a SAMchain.*

*Figure 3. SCtools performance. (a) Query time from a MultiChain stream depends only on the number of entries in that stream, i.e., it is not affected by the number of reads stored in other streams in the chain. (b) In a single-node SAMchain network, we measured the time performance of queryReads, queryDepth, queryDepth and MAPQ, and pileup for one-bp queries. Each module performs comparably, increasing linearly as the number of reads stored in the chain increases. (c) We measured the performance of queryReads for a SAMchain storing ~10,000,000 reads at different bin sizes. SAMchains with smaller bin sizes yield faster query times, but take longer to build. (d) We measured the effect of the range queried on performance time. Larger ranges are shown to yield longer query times. We showed the times of SAMchain (red) compared to a naive, single-stream implementation (blue). (e) We checked the output of each SCtools module compared to the equivalent SAMtools module.*

*Figure 4. VCFchain and query performance. (a) The total network storage used by a VCFchain (storing 6,458,146 variants) as a function of the number of nodes in the network. (b) The storage, time, and memory requirements of building and inserting data to a VCFchain in a single-node network as a function of the number of reads stored in the chain. Storage is compared to that used by a VCF file. (c) The time requirements for VCFquery as a function of the number of reads stored in the chain, compared to the retrieval time from a VCF file using BCFtools. (d) The output of VCFquery compared to that of BCFtools for a given read.*

*Figure 5. Schematic of storing .diff files in our blockchain implementation and the differences between the various implementations introduced in this study.*

*In “SCtools can query NGS properties directly from SAMchain”*

*As can be seen in Figure 3b, the time it takes to query a single genomic location using our different query mechanisms in a network with 10 million reads divided into 10,000 reads/stream is around 0.5 seconds. Note that as was shown in Figure 3a, the query time depends on the stream size; that is, if we have 10,000 reads per stream with 500 million reads, the query still takes 0.5 seconds because of the nested indexing scheme. As a comparison, we ran various queries with plaintext tool SAMtools12 using a BAM file with ~60 million reads. We found that pile up for a single location takes about 0.05 second; querying a depth of a single location takes about 0.02 second; and querying the reads mapped to a single location takes about 0.08 seconds. This shows that SCtools, on average, is about 10 times slower than SAMtools due to the added security. However, note that, in order to be able to perform these queries with SAMtools, one needs to sort and index the BAM files first, which is known to be a slow process. In SAMchain, reads are already stored in an indexed way based on their genomic coordinates and therefore there is no need for such pre-processing. However, one can think of sorting process as analogous to pushing the SAM data to the blockchain network as both processes are slow, but done only once.*

*In “SAMchain is the first proof-of-concept project to store raw genomic reads on a blockchain”*

*There are also non-blockchain infrastructures to store raw genomic data in a distributed and secure way. For example, IPFS is a distributed peer-to-peer network. Users in the network can store a file by its content address, and other peers in the network can find and request the file from any node using the distributed hash table implemented in IPFS. Although blockchain and IPFS are based on similar decentralization concepts, IPFS is a peer-to-peer file sharing system. By contrast, our SAMchain and SCTools are designed for the purpose of securely accessing and analyzing the data in a granular fashion, allowing users to index NGS data at the base-pair resolution. IPFS allows hashing the data at the file level and one would have to download the entire SAM file to be able to analyze and query base-pair resolution information. Another technology that allows sharing and analyzing of distributed genomic data is Arvados, which is an open-source platform for large scientific and biomedical data. It allows spinning of cloud computing instances using data that are decentralized to avoid expensive data transfers. It is a bioinformatics platform, in which an application such as SAMchain and SCTools can be implemented and run by instantiating cloud computing nodes. However, it is designed for the use of scientists requiring execution of Common Workflow Language-based scripting that might be difficult for clinicians and patients to adopt. Note that IPFS and Arvados, blockchain and SAMchain/SCtools are on different conceptual layers and because of this can not fully substitute for each other. In particular, while IPFS and Arvados are useful file systems and computing infrastructures that any application can be built on top of, SAMchain and SCtools are applications that are built on top of blockchain (Supplementary Figure 9)*

3.9 HPC setting

Reviewer’s comment In some ways the manuscript raises more questions then it answers. For

example blockchains can be distributed/federated/sharded. How do we benefit from these features, how does it impact mining information on an HPC? At this point the manuscript feels incomplete. I can see blockchains becoming a part of clinical solutions, but what is necessary to get there?

Author’s response Our system is completely based on a command-line operated Python codebase. We have tested it and it works well in an HPC setting. That means, as long as access is granted to an institution, researchers can easily connect and mine data using their existing HPC infrastructure, even using multithreading. Please note that there is no mining in private blockchains, safeguarding is provided by proof of authority. We have now added this detail to the paper.

In the clinical setting, the next steps would be creating a user-friendly app for patients and clinicians, such that it can be accessed via any device (e.g., mobile phones) such as how we access the EPIC system’s MyChart but in a more secure way. We have now discussed these steps in the discussion section of our paper.

*Excerpt from manuscript*

*In “SAMchain is user friendly and easy to use by users with no blockchain knowledge”*

*Furthermore, our system works well in a high performance computing (HPC) setting. This means, as long as access is granted, scientists can easily install the MultiChain client and SAMChain app and connect to an existing private blockchain network to mine genomics data by using their institution's existing HPC infrastructure.*

3.10 Access control

Reviewer’s comment Furthermore, the Multichain approach here is mostly about access control. So, how does that work for the individual? Will we create an app that allows them to say who can access their data? Or is it the sequencing organisation, such as 23andme or a hospital, that gives access to the data? That is probably not what the individual wants. I.e., more questions. GA4GH is cited - these folks are creating layers and layers of APIs for access control. How can we short-cut that work using Multichains? More questions that deserve answers from the experts!

Author’s response Since the owner of the data is the individual, we envisioned that the individuals are the deciders on who can access their data, which will hopefully cut down on the layers for access control. Here we created a proof-of-concept codebase that is envisioned to be implemented in the form of an app that people could even potentially use on their personal phones in the future.

*Excerpt from manuscript*

*In “Discussion”*

*Moreover, traditional access-control mechanisms such as dbGAP or EGA leave the research participant or patient outside of the decision-making process. Our private blockchain network reduces not only the risk of data corruption, but also non- permissioned access to private data and gives the control to the actual owner of the data.*

Reviewer’s comment Another point of note is that much of this work is based on the reference genome. Even though mapping and variant calling on the reference genome is still the dominant approach today, it is showing its short comings, and may well get replaced in the coming years. E.g. the following papers point out that new pangenome methods are emerging that are superior:

Eizenga, J. M. and Novak, A. M. and Sibbesen, J. A. and Heumos, S. and Ghaffaari, A. and Hickey, G. and Chang, X. and Seaman, J. D. and Rounthwaite, R. and Ebler, J. and Rautiainen, M. and Garg, S. and Paten, B. and Marschall, T. and Siren, J. and Garrison, E., Pangenome Graphs, Annual Review of Genomics and Human Genetics, 2020. Sherman, R. M. and Salzberg, S. L., Pan-Genomics in the Human Genome Era, Nature Reviews Genetics, 2020. ODGI: understanding pangenome graphs Andrea Guarracino, Simon Heumos, Sven Nahnsen, Pjotr Prins, Erik Garrison BioRXiv, 2021 doi: https://doi.org/10.1101/2021.11.10.467921

Just to elaborate a little on pangenomes:

With regard to the reference, how is it possible to capture the entire diversity of a species with an arbitrary single linear sequence? People are increasingly finding out it is not. A pangenome is a collection of genomes organized into a graph data structure to be used for holistic genetic analysis. It is hard to overstate the impact of moving from a sequence-based to a graph- based view of genomics. A traditional single sequence-based reference genome might be missing up to 10% of the genetic sequence in under-represented populations these missing sequences are the unexplored dark matter of the genetic universe. Today, all human DNA variants are identified comparing with this standard reference genome. Even though the `standard’ reference was a massive step forward at the time, it is now known to be biased and misrepresents whole populations: for example, 10% of African DNA sequence does not align to the current reference genome (see Sherman paper).

It would be interesting to bring pangenome methods to the block chain as they can be highly compressed (humans are very similar at the genomic level). Would it be possible to store and scale graphs this way? It may be worth touching on in the discussion or mentioning it as future work. the authors chose to use VCF and SAM which are highly tied to a reference genome-based approach.

Author’s response We thank the reviewer for this question. We are indeed aware of the graph-based pangenomes. We agree that the VCF and SAM are highly tied to the reference genome-based approaches.

To be able to assess how pangenomes would work with our system, we first have to see what kinds of new standard file formats will emerge from the pangenome use. As an initial thought, we agree with the reviewer that pangenome methods will even give us more compression opportunities and will make our system more tractable. We envision that instead of using a coordinate-based indexing system, we would use an edge-vertex based binning system to represent the reads in SAMChain. We have now added these thoughts and the references to the discussion.

*Excerpt from manuscript*

*In “Discussion”*

*Note that our system is based on the existing reference-genome based file formats. However, we envision that these file formats might be modified or replaced by other formats that use graph genomes and pan-genomes as references 32–34 . This will likely change the indexing and querying mechanisms that we propose in this study.*

3.11 Homomorphic encryption

Reviewer’s comment Likewise, the manuscript touches lightly on (homomorphic) encryption.

Since you cite a paper on GWAS, you might also want to check our contribution:

Private Genomes and Public SNPs: Homomorphic Encryption of Genotypes and Phenotypes for Shared Quantitative Genetics by Mott R, Fischer C, Prins P and Davies RW, Genetics (2020) Vol 215 p. 359-372, DOI: 10.1534/genetics.120.303153 (open access) These methods are very interesting and address the privacy protection that is missing in the blockchain approach.

Author’s response We thank the reviewer for this comment and for sharing with us their contribution. We find this work very interesting - we also published on genome analysis with homomorphic encryption (HE) last year. We have now incorporated these HE-based works into our discussion, mentioning the importance of privacy protection that might be added on top of the blockchain system we present here. However, we also would like to add that the overhead of HE on top of the overhead from blockchain could be a difficult problem to navigate if we were to build an HE-based blockchain data storage and query system.

*Excerpt from manuscript*

*In “Discussion”*

*For example, homomorphic encryption has recently been applied to many bioinformatics problems5,26–29 It enables direct computations of encrypted data within the public cloud or any other shared server, providing mathematical privacy guarantees. One could even encrypt the data homomorphically, allowing direct computations on the encrypted data 30 . However, this would add storage and computation overhead to the solution.*

3.12 ssh

Reviewer’s comment 14. Another thing that puzzles me is the suggestion of introducing ssh in the discussion. Without further explanation this is very confusing. Does the blockchain not have built-in access control? Why introduce ssh as a feature? And if it is one, maybe some examples will help. Besides, I don’t know about Yale, but most clinical environments I am aware of prevent the use of ssh unless you are already on an internal network (VPN)

Author’s response We thank the reviewer for raising this point. We understand that mentioning “ssh” is confusing as it is just how we accessed the nodes - we have now removed the mention of ssh from the manuscript as it is irrelevant to the underlying access mechanism.

However, it is true that the MultiChain platform does have built-in access control. We have now emphasized the ease of MultiChain built-in access control and how it works in the manuscript.

*Excerpt from manuscript*

*In “Private blockchain networks can help with control of data”*

*MultiChain, the technology that SAMChain is built upon, provides access control, in which the owner of the blockchain network can grant permission to a user and make the user part of the blockchain network. It also provides the option of granting only partial access to the chain.*

3.13 final comments

Reviewer’s comment

Sorry if I appear a bit critical. The short of it is that I like the paper — it puts a stake in the ground for blockchaining large data — but I think the authors should help readers a little more and, if possible, provide more context. From the current manuscript I am not convinced this technology will take off. At best it is an exploratory use of the MultiChain implementation which won’t scale (see below). Note that my PDF contains some notes and highlights. If you fail to see them use the free software Okular tool. And forgive me my English as I am not a native speaker.

Author’s response We thank the reviewer for their insightful comments and highlights. We find them very helpful and easy to understand. We would like to emphasize that this work is meant to be a proof of concept to guide the developers in a direction that was not explored before. Moreover, we do not think that we have a scalability issue here because 1- the genome is fixed in size and we showed that it can be stored on the blockchain with the current data storage options we have; 2- the cost of pushing data, although seems high, is a one-time cost and is attainable, as we showed with our new parallel system; 3- it is designed to hold a single human genome, which, as mentioned, has a fixed size; 4- for storing many genomes, we would create many SAMChains operated by the owner of the genomes. We have now added this to the discussion. We also plan on using this conceptual paper as the foundation to develop more usable, translational blockchain systems. We hope that our current changes to the manuscript will help the readers and provide the context needed.

As a note, neither the editor nor us were able to locate the said PDF - but we hope that we were able to fully address this reviewer's points.

*Excerpt from manuscript*

*In Discussion*

*Please note that, in our study, each private blockchain network corresponds to a single genome owned by the individual to which the genome belongs. In addition, a single genome will always have a fixed size (for example, 30X whole-genome sequencing will always have 500-600 million reads based on the read length). That is, genomes from millions of users translate into millions of private blockchain networks such that each network has to scale only to a fixed, single genome size. Therefore, we believe that neither storage nor the time to push the data (one time cost) will be an issue moving forward if this technology is widely adopted. Moreover, researchers can subscribe to multiple networks at once to query data in parallel from multiple genomes. In summary, we think that the scalability will not be a problem moving forward, because 1- the genome is fixed in size and we showed that it can be stored on the blockchain with the current data storage options we have; 2- the cost of pushing data, although seems high, is a one-time cost and is attainable, as we showed with our new parallel system; 3- it is designed to hold a single human genome, which, as mentioned, has a fixed size; 4- for storing many genomes, we would create many SAMchains operated by the owner of the genomes.*

Minor comments

3.14 wording

Reviewer’s comment 16. Some words perhaps do not belong in a science paper. I highlighted

the ones that caught my eye in yellow. For example the word ‘exciting’, if used, warrants further qualification.

Author’s response Thank you, we have now changed this wording.

3.15 open data initiatives

Reviewer’s comment 17. Open data initiatives, such as OpenSNP and the Harvard public genomes, are worth mentioning because they deal with genomic variants. If data is so sensitive, what justifies their existence? Can they benefit from a more secure setup? I think they can.

Author’s response We believe these initiatives only share genomic data that are fully broadly consented by the participants. Our system answers a different question: what if the participant would like to choose who should access their data? Moreover, even with publicly open data, there is still the issue of how we can keep the integrity of the data. Our system addresses that question. We have now made this more clear and mentioned these initiatives in the manuscript.

*Excerpt from manuscript*

*In Discussion:*

*For example, every time a piece of data is encrypted in a shared server or cloud, there is a potential for it to be corrupted, especially if the data are extremely large such as genomic data. Moreover, traditional access-control mechanisms such as dbGAP or EGA leave the research participant or patient outside of the decision-making process. Our private blockchain network reduces not only the risk of data corruption, but also non- permissioned access to private data and gives the control to the actual owner of the data. Blockchain provides immutability such that the data cannot be altered, whether intentionally or accidentally. This is an important aspect of the system not only for personal, private data but also for large-scale open genomic data. Large-scale open data such as those from the 1,000 Genomes Project or the Personal Genomes Project, especially in centralized data storage systems, are vulnerable to corruption and tampering.*

3.16 IPFS vs. blockchain

Reviewer’s comment 18. In the introduction the authors suggest this is the first time this approach of storing genome data in the block chain is taken. I think that is too strong a statement. I think combining IPFS hashes with blockchains is actually a better and more scalable solution. It may be worth pointing out that IPFS itself is not a block chain by some counts https://blockchain.works-hub.com/learn/blockchain-and- interplanetary-file-system-ipfs-cec4d but it guarantees immutable data and hashes. IPFS allows for sharding at the block level. The main problem with IPFS is that it is commonly used for public data. Even so, it is feasible to create private IPFS solutions though they are hard to share. So, this is where your block chain can shine. Perhaps we need a hybrid solution that actually works. Storing hashed values of files in the block chain looks like the way forward to me - and it is pointed out in the text that it is exactly what other existing solutions do. I.e. the next paragraph points out ‘many up and coming’ companies store genomic data elsewhere (probably referencing from a block chain). I think this text can be a bit clearer and clearer citations would be good. And what are ‘up and coming’ companies exactly?

Author’s response We had to move the information on these companies to the supplement for two reasons: 1- the space limitation for our paper as this is not a review paper; 2- most of the information about these companies are proprietary and the information we presented is what we could find publicly, i.e., we are worried about the accuracy. Regardless, as we mentioned above, our system answers a different question than the IPFS. IPFS does not allow for granular indexing; therefore, it cannot, for example, query a SAM file by a coordinate. There are genomics blockchain systems that store a pointer to the data that are stored on IPFS. However, in these systems, since data are kept outside the blockchain, they are still vulnerable to corruption.

*Excerpt from manuscript*

*In “SAMchain is the first proof-of-concept project to store raw genomic reads on a blockchain”*

*There are also non-blockchain infrastructures to store raw genomic data in a distributed and secure way. For example, IPFS is a distributed peer-to-peer network. Users in the network can store a file by its content address, and other peers in the network can find and request the file from any node using the distributed hash table implemented in IPFS. Although blockchain and IPFS are based on similar decentralization concepts, IPFS is a peer-to-peer file sharing system. By contrast, our SAMchain and SCTools are designed for the purpose of securely accessing and analyzing the data in a granular fashion, allowing users to index NGS data at the base-pair resolution. IPFS allows hashing the data at the file level and one would have to download the entire SAM file to be able to analyze and query base-pair resolution information. Another technology that allows sharing and analyzing of distributed genomic data is Arvados, which is an open-source platform for large scientific and biomedical data. It allows spinning of cloud computing instances using data that are decentralized to avoid expensive data transfers. It is a bioinformatics platform, in which an application such as SAMchain and SCTools can be implemented and run by instantiating cloud computing nodes. However, it is designed for the use of scientists requiring execution of Common Workflow Language-based scripting that might be difficult for clinicians and patients to adopt. Note that IPFS and Arvados, blockchain and SAMchain/SCtools are on different conceptual layers and because of this can not fully substitute for each other. In particular, while IPFS and Arvados are useful file systems and computing infrastructures that any application can be built on top of, SAMchain and SCtools are applications that are built on top of blockchain (Supplementary Figure 9).*

3.17 consensus mechanism

Reviewer’s comment 19. Do all blockchains require consensus verification? I do not think so. To me a block chain is an incremental set of hashes that can not be tampered with without noticing. If I am wrong or right, please expand on that for the layman. Consensus verification is a useful extra in my mind, but not core or even critical to a block chain. Verification and node discoveries are features of particular blockchain implementations. The paper should be super clear on these definitions.

Author’s response We thank the reviewer for this question. Briefly, a blockchain is a data structure consisting of a linked set of hashes (i.e., the hash of each block contains the hash of the previous block). It is true that this data structure can exist without any consensus verification. However, consensus verification becomes very useful in a blockchain network, as it is critical to the maintenance of the blockchain state (e.g., which transactions are included in a block, in what order, etc.). In a public blockchain, proof-of- work is often used as the consensus mechanism - miners have to exert some computational effort to prove that they are qualified to add a new block to the chain. However, in a private blockchain such as the one presented in this paper, proof-of-work is not used because there exists some element of trust in the group. Instead, the network can rely on other means of deciding who can add blocks, such as proof-of-authority, where permissioned accounts are able to add blocks in a round-robin scheme. In the paper, we clearly define the consensus mechanism used, and explain the distinction between public and private blockchains.

*Excerpt from manuscript*

*In Introduction*

*Private blockchains lack mathematical guarantees at the validation level, but still utilize the cryptographic safeguards and blockchain-specific data structures (e.g., Merkle trees) to make sure non-valid transactions are not added to the chain. Although proof-of-work is not used, transactions are verified using other consensus mechanisms. Private blockchain networks may be compared to distributed databases to illustrate their utility. Blockchain architecture has several advantages, as outlined by Kuo et al.11 . Briefly, blockchain excels in decentralized database management, immutability (provided by the hash of a new block containing the information of the blocks that come before it), data provenance, robustness, security, and privacy. Additionally, a private blockchain may be converted into a consortium blockchain, in which consensus is controlled by a pre- selected set of nodes (i.e., proof of work in a limited way), further boosting its security.*

3.18 scaling

Reviewer’s comment 20. Adding 10M reads (a typical NGS run for human) to the blockchain takes 55 hours. Wow. Even if that only happens once for a sequence set, I think it is a prohibitive amount of CPU time. Think about getting emergency clinical results after that lag. We should aim for less time instead of more between sequencing and results. Also CPU consumption is insensitive to current trends in climate change. There must be better methods for creating a genomic blockchain. I suggest MultiChain is not the right approach for sequence reads, though it is probably fine for efficiently stored variant calls. The discussion should bring that up. Present this paper like the pilot that it is and discuss what the remaining challenges are.

Author’s response We thank the reviewer for this comment. It is true that 55 hours may not be suitable for an emergency scenario, but there are many other scenarios in which accessing sequencing data securely is important. Moreover, we have now improved our storage time by 5-fold by implementing thread-level parallelism. Another point to keep in mind is that pushing the data to the network is a one time cost and considering genomes have fixed sizes, it is bounded. It is true that blockchain has been criticized from a climate change perspective, but this applies specifically to public blockchains utilizing proof-of-work and other computationally demanding consensus mechanisms. This does not apply to SAMChain, which does not require mining (see explanation above for public vs. private blockchains).

*Excerpt from manuscript*

*In “SAMchain provides a platform for storing next-generation sequencing (NGS) data in blockchain”*

*Since each read in a SAM file is independent and the streams to store the reads are automatically determined by our algorithm based on its coordinates, we can use multiple nodes to push the data in a SAM file in a parallel fashion. To this end, we parallelized our codebase and showed that we can improve data insertion time by 5-fold using 10 CPUs at the same time (Figure 2c). We also found that the 5-fold increase is the limit of the possible speedup because of the transaction latency in the blockchain. (This latency is due to safeguards such as proof of authority.) Furthermore, we tried to push 100,000,000 reads into the SAMchain, which took ~100 hours using 10 CPUs.*

**2^nd^ round**

**Reviewer 1**

I think this is considerably improved. I particularly appreciate the authors meticulous annotations of their responses. Their revised text and figures got at my primary issues, around scaling, usability and failure modes. They also addressed in text my concerns about the appropriateness of distributed database vs blockchain comparisons. I am very happy with this revision and think that this manuscript is ready for publication. I think that it will generate valuable and interesting discussion in the community.

**Reviewer 2**

The authors have done a great job of addressing all of my previous comments.

**Reviewer 3**

I just read the revised paper and I am happy that it is more focused and improved. The authors put a lot of work in. I think it is of interest to publish it as is.
